# Proteomic analysis reveals novel insights into tanshinones biosynthesis in *Salvia miltiorrhiza* hairy roots

**DOI:** 10.1038/s41598-019-42164-3

**Published:** 2019-04-08

**Authors:** Angela Contreras, Baptiste Leroy, Pierre-Antoine Mariage, Ruddy Wattiez

**Affiliations:** 1 Proteomics and Microbiology department, Research center for Biosciences, University of Mons, 20 place du Parc, Mons, 7000 Belgium; 2 Green2Chem, department of research, Ath, 7822 Belgium

## Abstract

*Salvia miltiorrhiza* is a medicinal plant highly appreciated by its content of tanshinones and salvianolic acids. Tanshinones are of particular relevance for their anti-oxidant, anti-tumoral and anti-inflammatory properties. Abiotic and biotic agents as silver nitrate and yeast extract have shown efficiently to stimulate tanshinone accumulation, but the underlying molecular mechanism remains essentially unknown. By using hairy roots as experimental material and the elicitors mentioned, were obtained up to 22 mg of tanshinones per gram of dry weight. Differential label-free quantitative proteomic analysis was applied to study the proteins involved in tanshinone biosynthesis. A total of 2650 proteins were identified in roots extracts, of which 893 showed statistically (p < 0.05) significant change in relative abundance compared to control roots, 251 proteins were upregulated and 642 downregulated. Among the upregulated proteins the predominant functional categories were metabolism (47%), stress defense (18%) and redox homeostasis (10%). Within the metabolism category, isoprenoid metabolism enzymes, cytochromes P450 and FAD-binding berberine proteins showed abundance profile linked to tanshinone concentration. The results presented here allowed to propose 5 new cytochromes P450 and 5 berberine enzymes as candidates to be involved into tanshinone biosynthesis, a novel finding that opens new avenues to improve tanshinone production through biotechnological approaches.

## Introduction

*Salvia miltiorrhiza* Bunge, commonly called danshen, is widely used in traditional Chinese medicine, to treat cardiovascular diseases and inflammatory processes^1–3^. This specie is regarded as a medicinal model plant because of its small genome, short life cycle, and amenability to stable genetic transformation^4^. Lipophilic diterpenoids (mainly tanshinones) and hydrophilic phenolic compounds (such as salvianolic acids; SaA) are the major active compounds found in its roots^5–8^. Tanshinones are a group of abietane diterpenes which includes dihydrotanshinone I (DHt), tanshinone I (TI), Cryptotanshinone (CTt) and tanshinone II A (TII). They all share relevant biological activities, such as anti-oxidant, anti-inflammatory, antibacterial, and even antitumoral properties^8,9^. The culture of *S. miltiorrhiza* hairy roots has been previously used to study the biosynthesis of tanshinones^10–13^ and other secondary metabolites^14–16^. However, low yields of tanshinones are typically obtained, as their production is associated with plant growth inhibition^12^.

Many pharmacological active compounds are naturally produced in response to different kind of environmental stresses as temperature changes, drought, pathogens attack and osmotic stress^3,16–19^, hence their production in tissue cultures could be stimulated by biotic and abiotic agents through a strategy called elicitation^20^. Elicitors such as methyl jasmonate (MeJA), yeast extract (YE), salicylic acid (SA) and AgNO_3_ (Ag^+^), used individually or in combination, can efficiently stimulate tanshinone accumulation in *S. miltiorrhiza* hairy roots^9,21,22^. Previous works have reported a 1.2 and 3.1 fold increase in the total tanshinone content over the control after 4 days of treatment using Ag^+^ (30 μM) and YE (100 mg L^−1^) elicitation respectively^18,23^. These treatments also increased SaA content^16^. The combination of both elicitors has shown to improve the tanshinone production in *S. miltiorrhiza* hairy root cultures much more significantly than individual treatments. The total tanshinones content can be increased by about 20% in comparison with a single elicitor^9^ and phenylpropanoid biosynthesis suppressed^8^. The effect of an elicitor on the secondary product yield is usually dependent on the dosage, time of elicitor addition to the culture and culture conditions^9,12^. Although significant progress has been made in elicitor uses and effects, little is known about the molecular regulation mechanism at cellular, physiological and biochemical level through which those stress agents induce the active compound accumulation.

Tanshinone precursors are biosynthesized via two pathways in plants: the mevalonate (MVA) pathway in the cytosol and the methylerythritol phosphate (MEP) pathway in plastids^8,24^. The 3-hydroxy-3-methylglutaryl coenzyme A reductase (HMGR) is the rate-limiting enzyme of the MVA pathway^25^. Geranylgeranyl diphosphate (GGPP), the universal precursor for the biosynthesis of diterpenoids, has been considered an important regulatory target in the tanshinone biosynthetic pathway^2,26^. The diterpenoid synthases: copalyl diphosphate synthetase (CPS) and kaurene synthase-like (KSL) catalyze GGPP conversion into miltiradiene, the mainstay of tanshinones^11,27,28^. Cytochrome P450 monooxygenases (CYP450s) play the most important role in the downstream modification of tanshinone biosynthesis. CYP76AH1, CYP76AH3, and CYP76AK1 have been shown to be involved in the catalytic biosynthesis of ferruginol, dihydroxy ferruginol, and dihydroxy sugiol, which are intermediate products^29–32^. Recently, 2OGD5, a member of 2-oxoglutarate-dependent dioxygenase (2OGD) superfamily was found to play a crucial role in the downstream biosynthesis of tanshinones following the hydroxylation of CYPs^33^. Despite of these significant progress, some oxidation and reduction steps involved in the tanshinones pathway are still unknown.

Proteomic has become one of the main approaches for analyzing and understanding biological systems^34,35^. Changes in gene expression at transcript level cannot always exactly show the changes at protein level^36^. Majority of proteomic analyses in plant roots have been focused on their response to different kind of stresses, such as, cold, drought, high salinity, or flooding stress, but only a few studies have focused on active compounds production^37^. In addition, most of those studies utilized the gel-based approaches resulting in the identification of the highest abundances proteins only. The proteomic study of the elicitor effects could improve the understanding of molecular mechanism to stress tolerance and secondary metabolism production. So far, only one such study has been reported by Wang *et al*.^12^ following the addition of YE and Ag^+^ to *S. miltiorrhiza* hairy roots during the first 5 days. Under this elicitation 64 proteins were identified showing activation of Ca2^+^/calmodulin signaling pathway, burst of reactive oxygen species and enhancing energy and carbon metabolism. However, the tanshinone biosynthetic pathway remains far to be entirely elucidated. Recent advances in analytical instrumentation and bioinformatics make label-free quantitation of proteins by liquid chromatography tandem mass spectrometry (LC-MS/MS) an efficient and robust platform to perform accurate relative quantitation of hundreds of proteins in many samples in a single experiment^35,37^. Use of this technique may significantly improve detectability of low abundant proteins such as transcription factors, kinases, and transport proteins among others.

In this study, the effects of different elicitors on tanshinone production in *S. miltiorrhiza* hairy root cultures were evaluated. LC-MS/MS proteomic approach was used to analyze the differentially expressed proteins in samples with unequal tanshinone concentration. The findings will be useful for further understanding of molecular mechanism of tanshinone biosynthesis and to enhance their production by metabolic engineering in the near future.

## Results and Discussion

### Effects of YE, Ag^+^, and YE + Ag^+^ elicitation on secondary metabolites production of *S. miltiorrhiza* hairy roots

**Figure 1:**
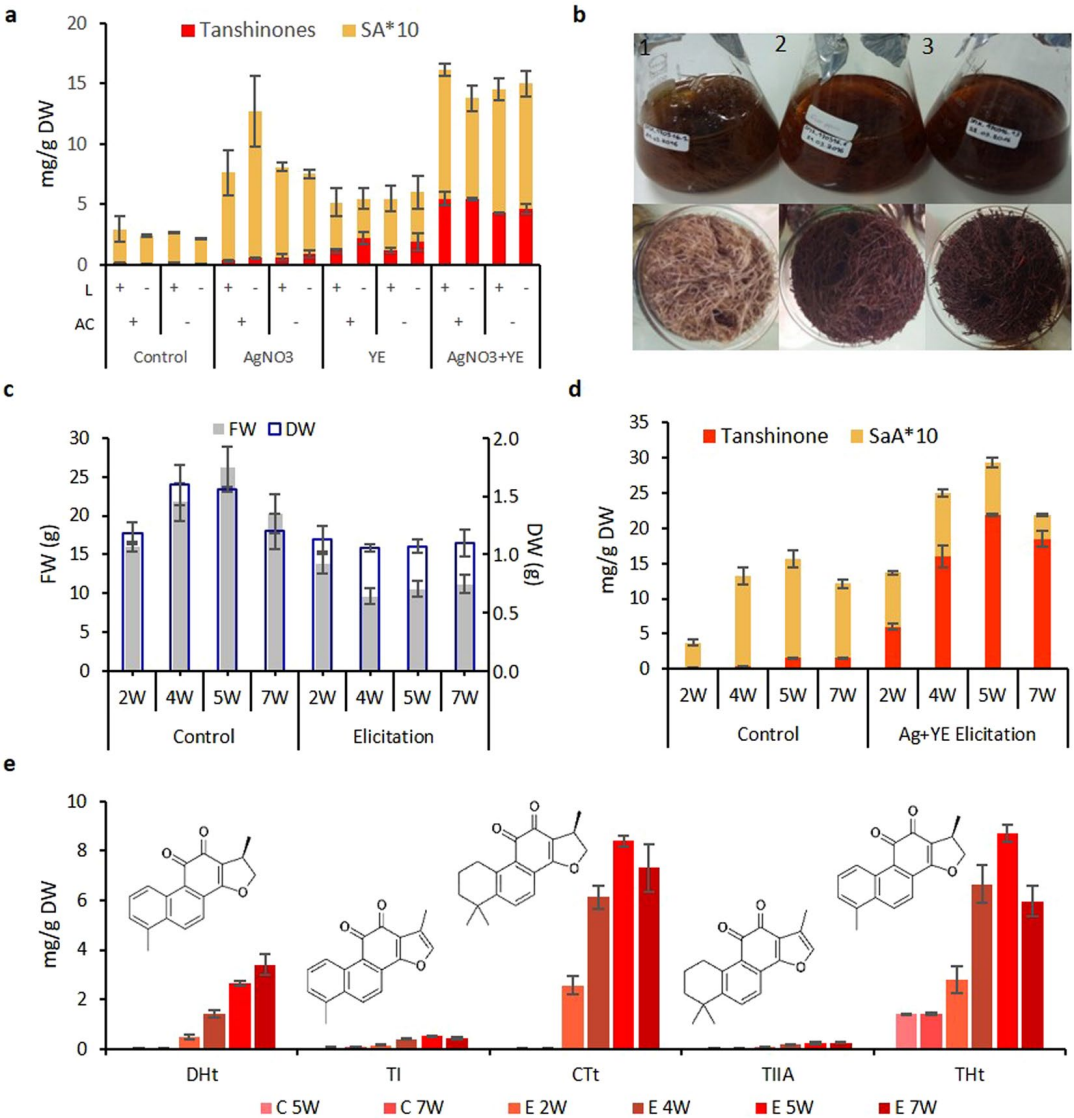
Elicitation process in *S. miltiorrhiza* hairy roots. (**a**) Effects of different elicitors and conditions (L = light and AC = activated carbon) in tanshinone and salvianolic acids production. (**b**) General appearance of medium and corresponding roots below under YE + Ag^+^ elicitation (1) control 5 weeks, (2) elicitation 4 weeks and, (3) elicitation 5 weeks. (**c**) Effect on growth and dry weight of YE + Ag^+^ elicitation for 7 weeks. (**d**) Changes on tanshinone and salvianolic acids content under YE + Ag^+^ elicitation for 7 weeks. (**e**) Changes in the profile of main tanshinones of *S. miltiorrhiza*; DHt, dihydrotanshinone; TI, tanshinone I; CTt, cryptotanshinone; TIIA, tanshinone IIA; THt, tetrahydrotanshinone.

A preliminary study based on roots survival and red coloring after 2 weeks of treatment, (data not shown), revealed that 1 g L^−1^ YE and 0.41 mM Ag^+^ were optimal doses for elicitation in our conditions. The tanshinone accumulation and SaA content were registered in presence or absence of light (L), and activated carbon (AC) using the elicitors individually and mixed. Both elicitors increased the content of tanshinones (Fig. 1a) which was not detectable in samples without elicitation (control). Combination of YE + Ag^+^ exerted the most effective induction on tanshinone biosynthesis. SaA were also induced by Ag^+^ and YE + Ag^+^ and slightly by YE. The light and activated carbon treatment did not show significant changes in tanshinones accumulation under Ag^+^ elicitation, however, darkness increased significantly the tanshinone content under YE elicitation and, activated carbon did under YE + Ag^+^ elicitation. As a result, YE + Ag^+^ as elicitor, no light and activated carbon as cultivation conditions were selected for further experiments. Activated carbon has been reported as a potent agent to improve some secondary metabolite, increasing the taxane synthesis^38^, cell growth and development^39^. These effects probably could be due to its irreversible adsorption of inhibitory compounds in the culture medium and substantially decreasing the toxic metabolites and phenolic exudate accumulation. It is well known that light can affect the metabolite production, low intensity and absence of light was reported to increase ferruginol biosynthesis^40^, an intermediary in tanshinones biosynthesis, and reduce levels of phenolics^41^. Our results confirmed the combination of biotic elicitor (YE) with abiotic elicitor (Ag^+^) is more effective to produce tanshinones than a single elicitor; observations previously reported in *S. miltiorrhiza* hairy roots by Wang *et al*.^9^ and other goups^8,42^. Furthermore, darkness and activated carbon presence could improve tanshinones contents. Under our conditions, 5 mg g^−1^ DW of tanshinones were obtained after 2 weeks-elicitation. This concentration was comparable to that reported by Cheng *et al*.^42^ using 2.5 g L^−1^ YE + 100 µM Ag^+^ after 5 days-elicitation, but much more higher than those reached by Wang *et al*.^12^ after 5 days of elicitation using 100 g L^−1^ YE and 3 mM Ag^+^ (0.7 mg g^−1^ DW), Kai *et al*.^8^ after 9 days using 100 mg L^−1^ YE and 0.03 mM Ag^+^ (2.08 mg/g DW), Ge *et al*.^23^ after 8 days using 30 µM Ag^+^ (0.96 mg g^−1^ DW) and 0.1 g L^−1^ YE (2.48 mg g^−1^ DW).

### Effects of YE + Ag^+^ elicitation on growth of *S. miltiorrhiza* hairy roots

To evaluate the impact of YE + Ag^+^ elicitation on the root biomass production, the fresh weight (FW) and dry weight (DW) were measured along the elicitation time starting on the second week coinciding with the coloring appearance due to the presence of thanshinones in the medium and followed for 7 weeks. In comparison with the respective control samples (Fig. 1c), FW was slightly reduced in the first 2 weeks of treatment and clearly reduced at 4, 5 and 7 weeks, down to 50%. However, the DW was not affected for the treatment, hence the ratio between FW and DW was much higher in samples under elicitation and the final biomass was around the same in all samples. It suggests a reduction of water content as effect of the YE and Ag^+^ treatment. Similar results were observed by Wang *et al*.^9^ after treatment with 25 μM of Ag^+^, Cd_2_^+^ and 100 mg L^−1^ of YE in which the enhanced tanshinone production was associated with a notable suppression of the biomass growth. Xing *et al*.^22^ reported, after Ag^+^ treatment of *S. miltiorrhiza* hairy roots, a slightly depressed growth after day 9, but a dry weight (DW)/fresh weight (FW) significantly increased due to the reduction of water content. Li *et al*.^26^ described YE as the most effective biotic elicitor for its intensive regulation on secondary metabolites and a concentration of 200 mg L^−1^ in *S. castanea* Diels *f. tomentosa* Stib. hairy roots as the most recommendable elicitor for dry matter accumulation and active tanshinone enhancement. Our results indicated that in *S. miltiorrhiza* hairy roots the combination of 1 g L^−1^ YE and 0.41 mM Ag^+^ is efficient in increasing the tanshinones concentration without significant variation in biomass.

### Effects of YE + Ag^+^ elicitation on tanshinones production of *S. miltiorrhiza* hairy roots

In this work, four main tanshinones, DHt, TI, CTt and TII, were quantified at 2, 4, 5 and 7 days after elicitation using HPLC. A unknown highly intense peak was observed in our chromatogram (Supplementary Fig. S1). This compound was analyzed by mass spectrometry giving precursor [M + H]^+^ ions at m/z 281 and CID fragment at m/z 263, 253, 235 and 221. Based on previous tanshinone identification works^43–45^, it was tentatively identified as 1, 2, 15, 16-tetrahydrotanshinone I (THt), a compound named trijuganone B, and included into the subsequent HPLC analysis. Under YE + Ag^+^ treatment, tanshinone content was significantly increased to 6 mg g^−1^ DW after 2 weeks-elicitation and 15 mg g^−1^ DW after 4 weeks. The maximum content was 22 mg g^−1^ DW reached after 5 week-elicitation (15-fold of the control) and a slight decrease was registered after 7 weeks-treatment (Fig. 1d). The main tanshinones affected by YE + Ag^+^ were DHt, CTt and THt, their content on 5 weeks-treatment reached 2.64 mg g^−1^ DW (264-fold the control), 8.39 mg g^−1^ DW (419-fold the control) and 8.69 mg g^−1^ DW (6-fold of the control), respectively (Fig. 1e). In control samples, tanshinones were not detected until 5 and 7 weeks in a low production of 1.5 mg g^−1^ DW confirming their production in response to stress^13^. The doses used and the time of elicitation evaluated in this work allowed a 40 times increase in the level of tanshinone concentrations compared with previously reported works under elicitation with YE and Ag^+^ which the maximum amount obtained was 5 mg g^−1^ DW^42^. Unlike the other tanshinones analyzed here, DHt is the unique tanshinone for which we observed an increased abundance on 7 weeks-treatment when the global tanshinone accumulation is reduced. On the other hand, THt is the only tanshinone for which abundance increased without elicitation on 5 and 7 weeks. These results suggest that THt could be an early responder to stress, while DHt would be involved in long lasting resistance to stress.

### Effects of YE + Ag^+^ elicitation on salvianolic acids production of *S. miltiorrhiza* hairy roots

HPLC analysis showed high SaA concentration in control samples, from 35 mg g^−1^ DW at 2 weeks to 140 mg g^−1^ DW on 5 weeks. Under YE + Ag^+^ treatment, SaA were increased in the first weeks of treatment to 77 mg g^−1^ DW (2-fold of the control), but then, they showed a continuous reduction, 90 mg g^−1^ DW (1.4-fold less than control), 74 mg g^−1^ DW (1.9-fold less than control) and 34 mg g^−1^ DW (3-fold less than control) on 4, 5 and 7 weeks respectively (Fig. 1d). Compared with the induction of tanshinones, YE and Ag^+^ could also enhance SaA accumulation in *S. miltiorrhiza* on the first 4 weeks of treatment, but they were reduced along the elicitation, showing a negative correlation with tanshinone concentration. Phenolic acids have demonstrated to be more sensitive than tanshinones during the plant’s response to YE and Ag^+^ stress^12^, but in most cases treatment duration has not been higher than 6 days. In Ag^+^ treatment SaA increase 1.3-fold on Day 6 after treatment^22^. Nonetheless, Wang *et al*.^12^ observed under YE and Ag^+^ treatment, that the level of the SaA dropped in the first 8 hours and then gradually increase again after 5 days. Our results confirmed the increase of SaA after a few days of elicitation but after long term elicitation the trend revert and SaA decrease.

### Overview of the quantitative proteomics analysis

**Figure 2:**
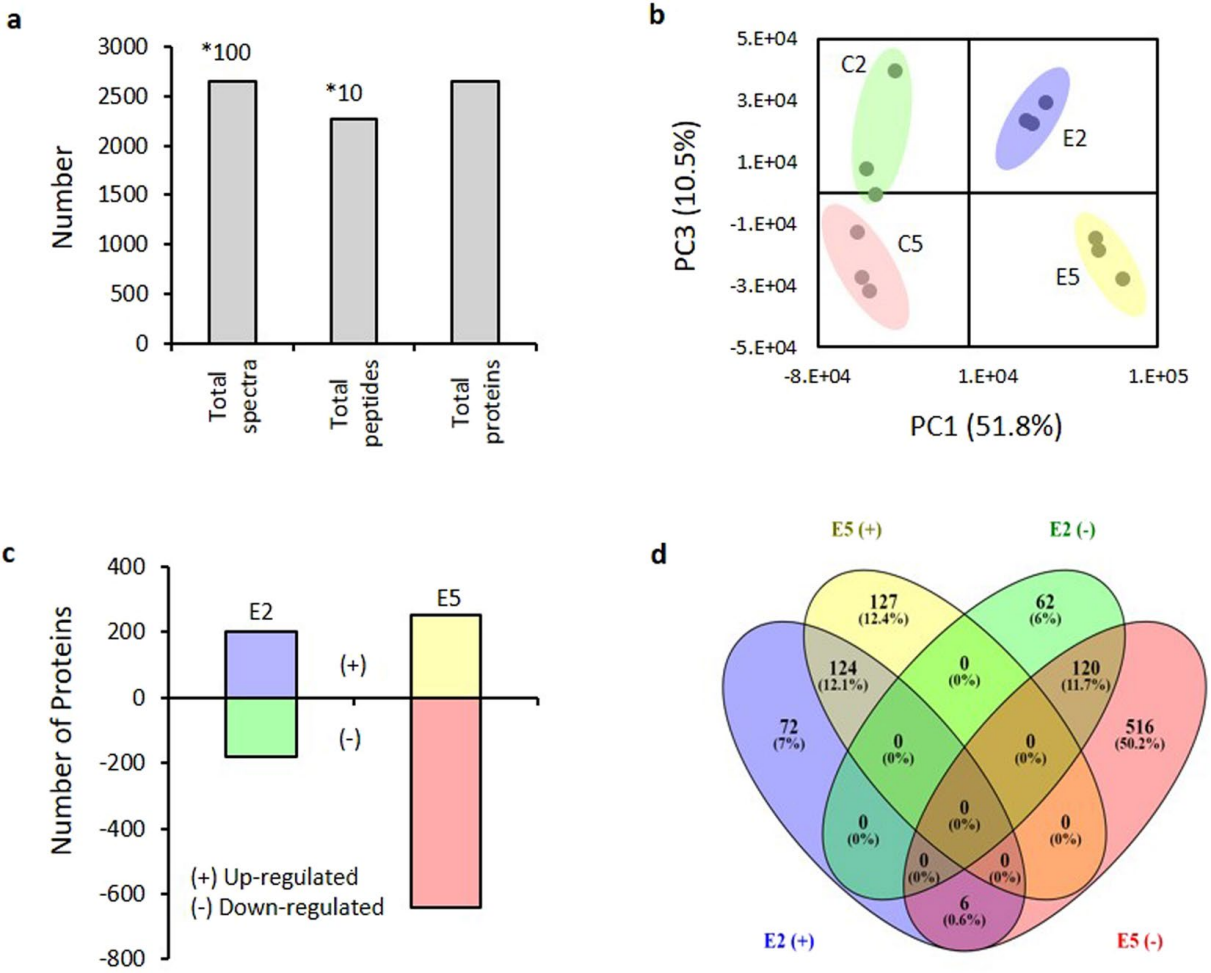
Overview of the quantitative proteomics analysis. (**a**) Spectra, peptides and proteins identified from LC/MS-MS proteomics by searching against our *S. miltiorrhiza* database at critical False Discovery Rates (FDR) of 1%. (**b**) Principal component analysis (PCA) performed using Markerview. Results represented based on changes in protein regulation. Axes show clear differentiation among samples containing 6 mg g^−1^ of tanshinones (E2) and 22 mg g^−1^ of tanshinones (E5) and their respective controls B2 and B5. A different colored circle represents each sample group (ellipses drawn as a guide to the eye). (**c**) The number of up and down regulated proteins of *S. miltiorrhiza* at low and high tanshinones concentration obtaining by comparison with their respective control samples. (**d**) Venn diagram showing the overlap between up- and down-regulated proteins of *S. miltiorrhiza* at low and high tanshinones concentration.

For the protein identification, a *S*. *miltiorrhiza* proteomic database was built in this work. The database was based on 34.575 protein sequences obtained from Chinese Herbal Plant Genome Databases^1^, 47012 protein coding RNAs from Danshen Transcriptional Resource Database^4^, which were translate into protein using ORFPredictor software^46^ and 1.631 protein sequences including 136 P450 Cytochromes from National Center for Biotechnology Information^47^. The redundant sequences were removed, and annotation was performed as described in methods section. A total of 52.092 protein sequences were considered in this study and assigned to 35 gene ontology categories (Supplementary Fig. S2). Gel-free LC-MS/MS proteomic analysis was applied to investigate the proteins involved in the biosynthesis of tanshinones. Samples under YE + Ag^+^ treatment of 2 and 5 weeks (E2, E5) were selected based on tanshinone concentration, 6 mg g^−1^ DW and 22 mg g^−1^ DW respectively, as well as their individual control (C2, C5). Three biological replicates were used for the analysis. A total of 2.650 proteins were identified from 22.679 unique peptides (Fig. 2a). Principal component analysis (PCA) of the processed datasets was performed to visualize the clustering trends among the samples selected. In the score plot of PCA, the different groups C2, C5, E2 and E5 were well differentiated (Fig. 2b). Using a threshold of a 1.5-fold change in abundance (±, compared to the respective control), identification with at least 2 peptides and a *p*-value lower than 0.05, 384 proteins were found to have a significant change in abundance in E2 (202 proteins upregulated and 182 downregulated) in contrast to 893 proteins altered in E5 (251 proteins up-regulated and 642 down-regulated), the down-regulation of several proteins being the major difference between E2 and E5 (Fig. 2c). The Venn diagram (Fig. 2d; Supplementary Data S1) indicated at 72 unique proteins induced in E2, 124 proteins induced in both treatments E2 and E5 and 127 unique proteins in E5. 62 and 516 unique down-regulated proteins were identified in E2 and E5 respectively and 120 shared in both conditions.

### Functional classification of proteins

**Figure 3:**
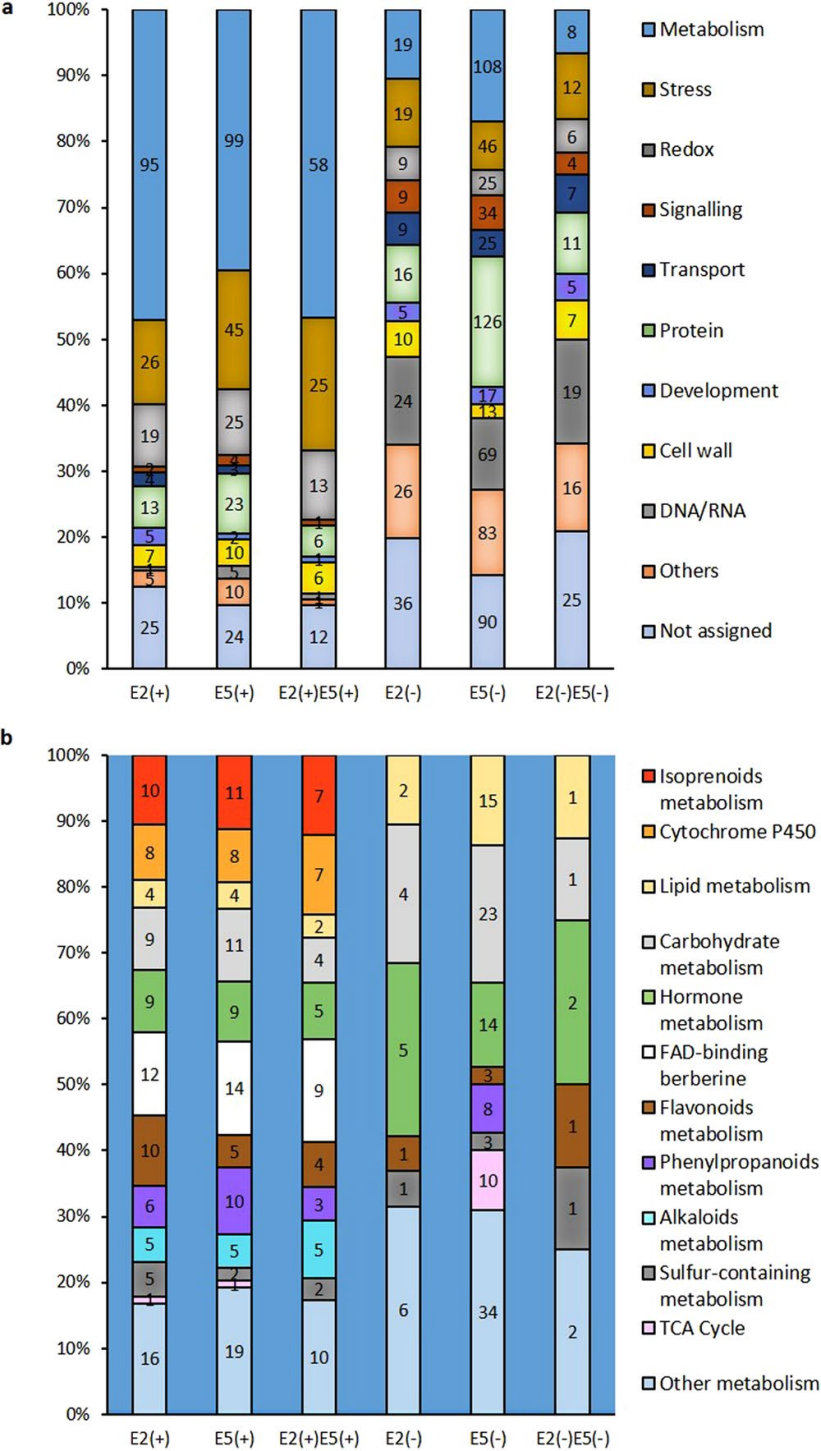
Comparison of the proteomic variations in samples with different tanshinone concentration at biological-level. (**a**) Bar chart showing the percentage of proteins upregulated (+) and downregulated (−) per GO category for the samples containing 6 mg g^−1^ of tanshinones (E2) and 22 mg g^−1^ of tanshinones (E5) and the protein shared in both samples. Inside de bars, number of proteins identified. (**b**) Bar chart specifying the percentage of proteins and categories included into metabolism.

The proteins identified were sorted manually into 11 gene ontology categories (Supplementary Table S1). The largest up-regulated category was “metabolism” with 95 proteins (47%) in E2, 99 proteins (39.6%) in E5, of which 58 are present in both points of elicitation; followed by “stress” with 26 proteins (12.9%) in E2, 45 proteins (18%) in E5 including 25 proteins in common; and “redox” with 19 proteins (9.4%) in E2, 25 proteins (10%) in E5 and 13 proteins in common (Fig. 3a). These results confirmed the influence of stress defense and redox homeostasis in the production of secondary metabolite in *S. miltiorrhiza* hairy roots. Down-regulated proteins were found distributed along all categories in E2 without special predominance. Despite E5 contains 460 down-regulated proteins more than E2, it conserves the profile for most categories, except for “metabolism” and “protein” categories, representing 17% and 20% of proteins, respectively.

Within the metabolism category (Fig. 3b and Supplementary Table S2), 12 subcategories were considered for this analysis: “isoprenoids metabolism” related with the synthesis of tanshinones precursors, “cytochrome P450” involved in structural and secondary metabolite diversity^48^, “FAD-binding berberine” a family recently reported their involvement in oxidative reactions^49^, “flavonoids metabolism” and “phenylpropanoids metabolism” related with SaA synthesis, among others. In general, the proteins up-regulated in E2 and E5 samples were equivalently distributed with the same percentage along the different categories. However, in E5 the number of proteins involved in flavonoid metabolism decreased, coinciding with the reduction of SaA observed. Despite most categories exhibited both up and down-regulated proteins, 3 categories were found with only up-regulated proteins, such *i.e*. “isoprenoids metabolism”, “cytochrome P450” and “FAD-binding berberine”. As opposed to E2 samples, E5 showed more proteins down-regulated than up-regulated ones in the categories of “lipid metabolism”, “carbohydrate metabolism”, “hormone metabolism” and “TCA cycle”.

### Expression profile of the proteins involved in terpenoid biosynthesis

**Figure 4:**
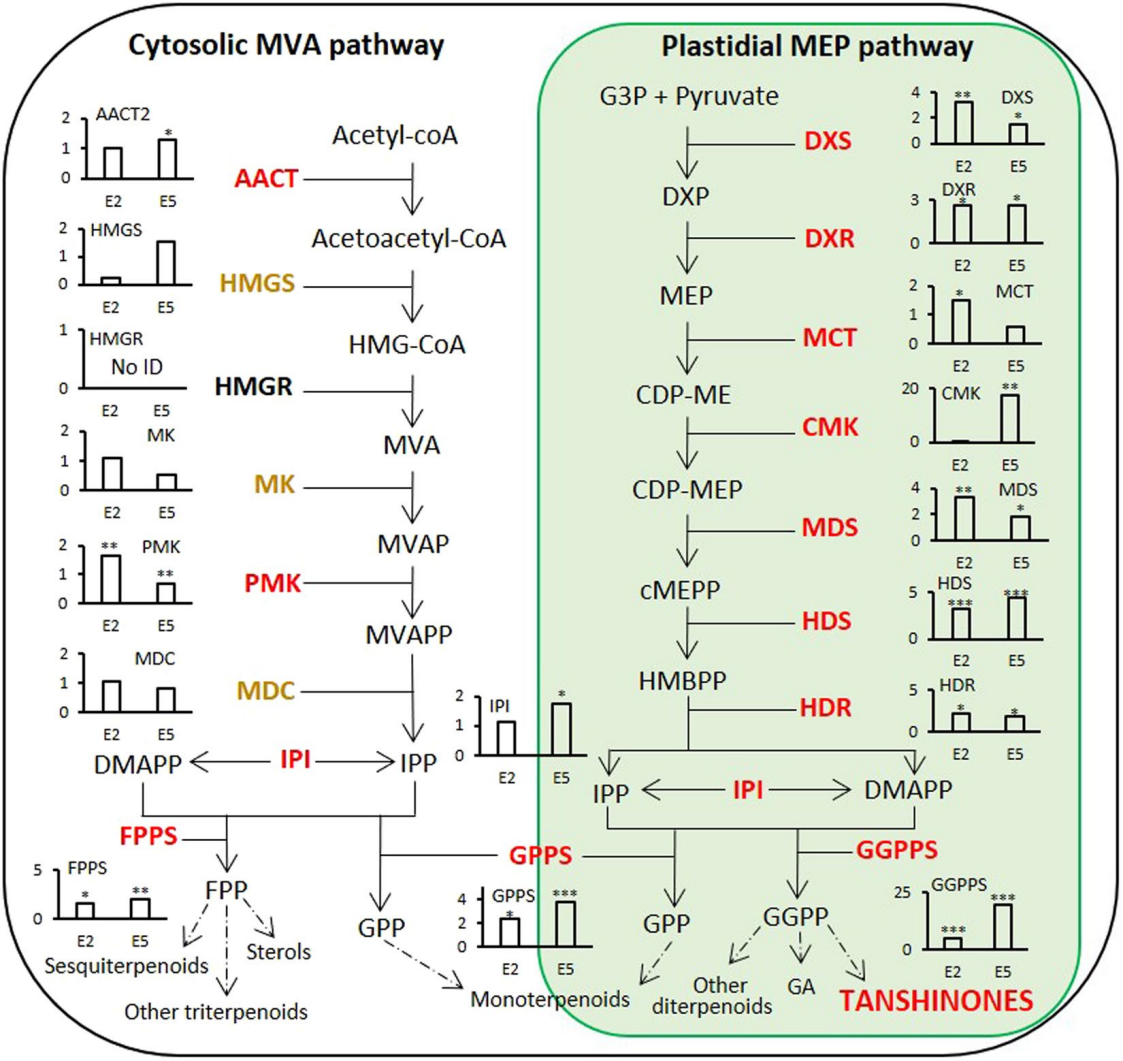
Overview of the proposed biosynthetic pathway of Terpenoids in *S. miltiorrhiza*. Graphs inside the pathway indicate the expression levels of the corresponding pathway proteins in hairy roots (the y-axis presents relative expression level; data represent means from three independent biological replicates) under different growth time points and elicitation time. Upregulated proteins are indicated in red and yellow ones did not present significant changes. Proteins in black were not identify in the analysis. The asterisk indicates significant differences at ***p ≤ 0.001, **p ≤ 0.01 and *p ≤ 0.05 using one sample T-test analysis. Abbreviations: AACT, acetyl-CoA C-acetyltransferase; CDP-ME, 4-(cytidine 5′-diphospho)-2-C-methyl-D-erythritol; CDP-MEP, 2-phospho-4-(cytidine 5′-diphospho)- 2-C-methyl-D-erythritol; CMK, 4-(cytidine 5′-diphospho)-2-C-methyl-D-erythritol kinase; DMAPP, dimethylallyl diphosphate; DXP, 1-deoxy-D-xylulose 5-phosphate; DXR, 1-deoxy-D-xylulose 5-phosphate reductoisomerase; DXS, 1-deoxy-D-xylulose 5-phosphate synthase; FPP, farnesyl diphosphate; FPPS, farnesyl diphosphate synthase; G3P, glyceraldehyde 3-phosphate; GA, gibberellin; GGPP, geranylgeranyl diphosphate; GGPPS, geranylgeranyl diphosphate synthase; GPP, geranyl diphosphate; GPPS, geranyl diphosphate synthase; HDR, 4-hydroxy-3-methylbut-2-enyl diphosphate reductase; HDS, 4-hydroxy-3-methylbut-2-enyl diphosphate synthase; HMBPP, 4-hydroxy-3-methylbut-2-enyl diphosphate; HMG-CoA, 3-Hydroxy-3-methylglutaryl-CoA; HMGR, hydroxymethylglutaryl-CoA reductase; HMGS, hydroxymethylglutaryl-CoA synthase; IPI, isopentenyl diphosphate isomerase; IPP, isopentenyl diphosphate; MCT, 2-C-methyl-D-erythritol 4-phosphate cytidylyltransferase; MDC, mevalonate pyrophosphate decarboxylase; MDS, 2-C-methyl-D-erythritol 2,4-cyclodiphosphate synthase; cMEPP, 2-C-methyl-D-erythritol 2,4-cyclodiphosphate; MEP, 2-C-methyl-D-erythritol 4-phosphate; MK, mevalonate kinase; MVA, mevalonate; MVAP, mevalonate-5-phosphate; MVAPP, mevalonate-5- diphosphate; PMK, 5-phosphomevalonate kinase.

Most of the MVA related proteins did not significantly change their abundance at any analyzed point of elicitation, except Acetyl-CoA C-acetyltransferase (AACT) which exhibited slightly higher abundance than control in E5 samples, and 5-phosphomevalonate kinase (PMK) in E2 samples (Fig. 4). On the other hand, all proteins of MEP pathway showed significant upregulation after elicitation, confirming that the synthesis of tanshinones derives predominantly from MEP pathway^48,50^. Among those enzymes, 1-deoxy-D-xylulose 5-phosphate synthase (DXS), 2-C-methyl-D-erythritol 4-phosphate cytidylyltransferase (MCT), and 2-C-methyl-D-erythritol 2,4-cyclodiphosphate synthase (MDS) were all three significantly upregulated in E2 samples and their upregulation decreased or even disappeared in samples of E5. In the case of 1-deoxy-D-xylulose 5-phosphate reductoisomerase (DXR), 4-hydroxy-3-methylbut-2-enyl diphosphate reductase (HDR) and, 4-hydroxy-3-methylbut-2-enyl diphosphate synthase (HDS), E2 and E5 samples exhibited similar abundance profile. However, a very high abundance was observed in E5 samples, coinciding with the maximum tanshinone concentration observed, for the enzymes 4-(cytidine 5′-diphospho)-2-C-methyl-D-erythritol kinase (CMK, 18-fold of the control), as well as for geranylgeranyl diphosphate synthase (GGPPS, 20-fold of the control), this last enzyme being responsible for the synthesis of GGPP, the precursor of tanshinones^21^. Our results suggest that CMK and GGPPS abundance increase along the elicitation and advise HDS as an additional enzyme regulated by YE + Ag^+^ treatment. In previous works, the overexpression of GGPPS associated to the high production of tanshinones in *S. miltiorrhiza* hairy root pointed that GGPPS is an important regulatory site due to its location in the tanshinone biosynthetic pathway^51^. The overexpression of HMGR, DXS and GGPPS-DXS have been also reported in transgenic hairy root lines, significantly enhancing tanshinone production^10,30,52^. In double overrexpressors containing GGPPS and DXS, the tanshinones level was reached to 12.93 mg g^−1^ DW^10^, which is the highest tanshinone content that has been achieved through genetic engineering. Remarkably, our elicitation strategy allowed higher production yield since tanshinones reached the level of 22 mg g^−1^ DW.

### Profile of the proteins involved in proposed biosynthetic pathway of tanshinones

**Figure 5:**
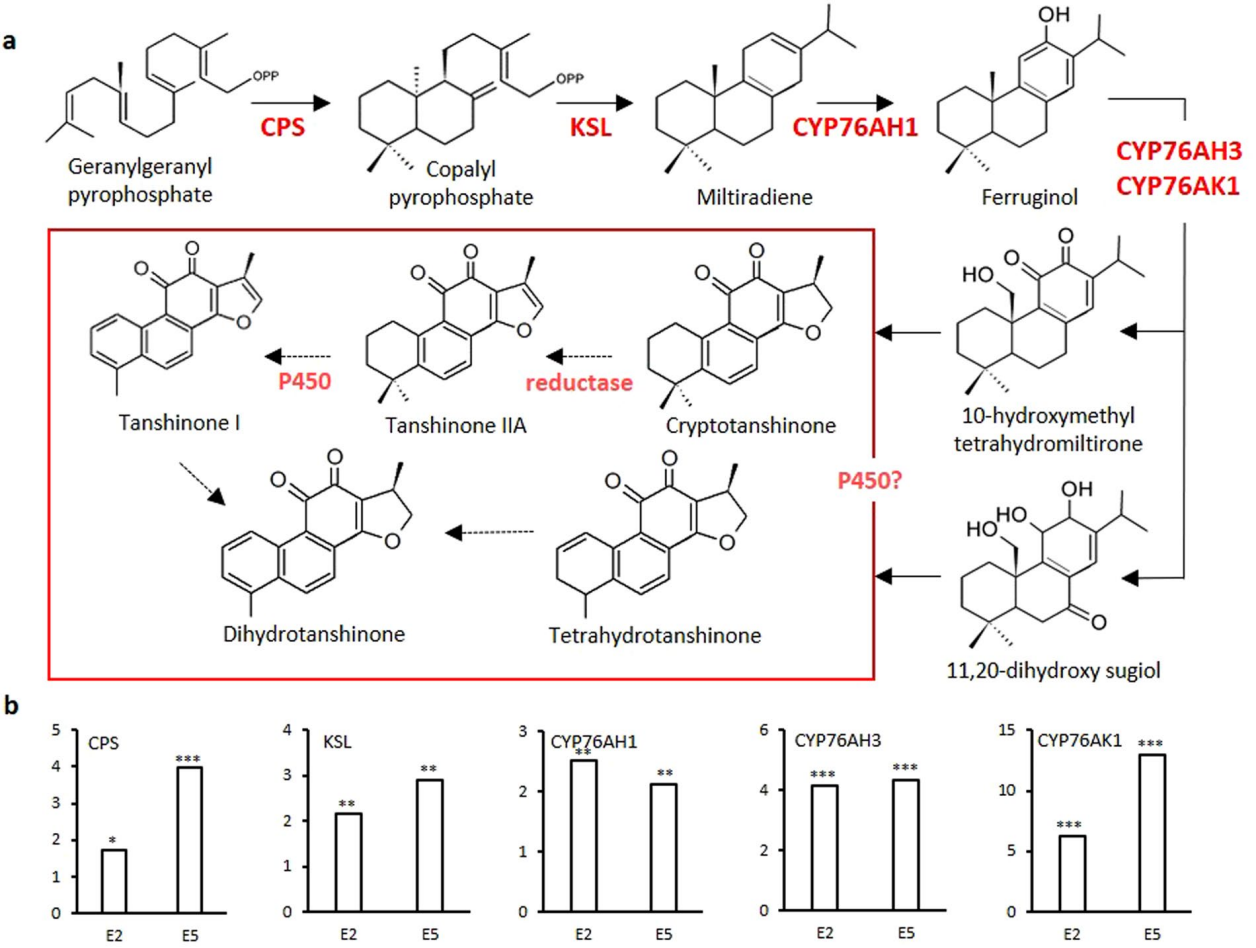
Biosynthetic pathway of Tanshinones in *S. miltiorrhiza*. (**a**) Overview of proposed biosynthetic pathway of tanshinones. Solid arrows indicate established relationships; dashed arrows indicate hypothetical relationships. (**b**) Graphs indicate the expression levels of the corresponding pathway proteins in hairy roots (the y-axis presents relative expression level; data represent means from three independent biological replicates) with low and high tanshinones concentration. All known proteins were induced in our analysis. The asterisk indicates significant differences at ***p ≤ 0.001, **p ≤ 0.01 and *p ≤ 0.05 using one sample T-test analysis. Abbreviations: CPS, copalyl diphosphate synthase; KSL, kaurene synthase-like; CYP76AH1, ferruginol synthase.

In our analysis CPS and KSL, CYP76AH1, CYP76AH3 and CYP76AK1 enzymes were significantly upregulated in both E2 and E5 samples (Fig. 5a,b). Their abundance, except CYP76AH1, was positively correlated with the increase of tanshinones production. The highest upregulation was showed by CYP76AK1, in agreement with the result observed by Guo *et al*.^32^, who using an RNAi approach indicated that suppression of CYP76AK1 led to significantly lower concentrations of TIIA, CTt and TI. The high abundance of this cytochrome and its upregulation linked to tanshinone production suggest an important role in the downstream tanshinone pathway. In previous works, CPS and KSL mRNA levels were also observed to be gradually increased after polysaccharide treated used to stimulate tanshinone production^53^, in contrast RNAi knockdown of *Sm*CPS expression in hairy roots reduced significantly DHt and CTt accumulations^54^ and recently, the use of CRISPR/Cas9 to obtain homozygous lines knock of the same *Sm*CPS revealed that tanshinones are completely missing^29^. Despite the significant progress in analyzing the tanshinone pathway, many enzymes responsible for oxidation steps involved in the biosynthesis of downstream metabolites remain to be identified. Therefore, we used the abundance profiles obtained in this proteomic analysis to find out new candidates involved in tanshinones biosynthesis.

### Possible proteins candidates involved in biosynthetic pathway of tanshinones

**Figure 6:**
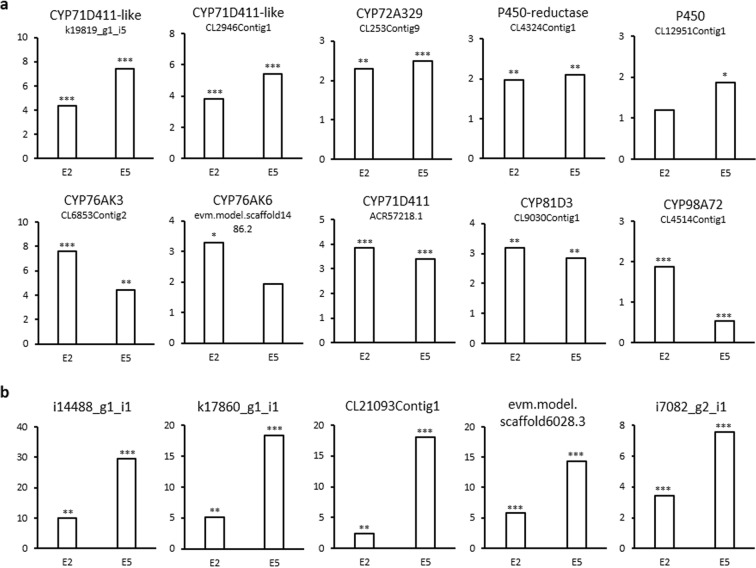
Protein candidates in tanshinone pathway of *S. miltiorrhiza*. (**a**) Cytochromes P450 identified in our proteomic analysis. (**b**) FAD-binding berberine proteins highly upregulated. The asterisk indicates significant differences at ***p ≤ 0.001, **p ≤ 0.01 and *p ≤ 0.05 using one sample T-test analysis.

CYP450s could likely play important roles in the process of oxidation/oxygenation and given their importance in the intermediates production for tanshinones biosynthesis^48^, the expression for all CYP450s found in the present work were analyzed. 10 proteins were identified as CYP450 (Fig. 6a), 5 of which are potentially involved in tanshinone biosynthesis based on their expression increase in E5 samples. Their annotation was based in the comparison with CYP450s protein sequences previously reported in GenBank. Among the CYP450s candidates, 2 proteins were named as CYP71D411-like, K19819_g1_i5 and CL2946Contig1, and both presented the highest upregulation observed among the orphan CYP450, 7.5 and 5.4-fold of the control, respectively. K19819_g1_i5 presented 65% identity with CYP71D411 (GenBank: ACR57218.1) and 63% with CYP71D410 (GenBank: AJD25163.1) from *S. miltiorrhiza*. CL2946Contig1 presented 99% of identity with AGN04216.1 from GenBank, annotated as CYP450 without any specific classification. CL2946Contig1 also presented 65% identity with CYP71D411 (GenBank: ACR57218.1). Despite both proteins showed the same identity with CYP71D411, the differences observed in their alignment claims for considering them as two different CYP450 enzymes (Supplementary Fig. S3). CYP71D411 has been related to the secondary metabolism of monoterpenoids^55^; however, in our functional annotation it was related to the secondary metabolism of flavonoids, hence with SaA and phenolic compounds. In our proteomic analysis CYP71D411 decreases in E5 samples, which would coincide with the reduction of SaA observed. The higher upregulation of K19819_g1_i5 and CL2946Contig1 in E5, diverging from CYP71D411 abundance profile, permitted us to suggest another function for these enzymes potentially related to tanshinone biosynthesis. The protein sequence CL253Contig9 showed a similar upregulation in samples E5 and E2, presented 90% identity with CYP72A329 from *S. miltiorrhiza* (GenBank: AJD25170.1) and was annotated as secologanin synthase-like, a precursor of monoterpenes. Unfortunately, the actual function of CYP72A329 is currently unknown, and its annotation is purely theoretical. CL45324Contig1 also showed a constant abundance profile in E5 and E2 samples and was annotated as CYP450 reductase 2. Its function is unknown, but reductases seem to be required for downstream step in tanshinone biosynthesis. CL2951Contig1 was significantly upregulated only in samples E5 and presented 77% identity with CYP71D329 from *Plectranthus barbatus*. This plant belongs to the same family as *S. miltiorrhiza* (Lamiaceae) and abietane diterpenes are one of its main active compounds produced^56^ making CL2951Contig1 an additional candidate. On the other hand, we identified the sequences CL6853Contig2 (92% identity with CYP76AK3 from *S. miltiorrhiza*) and evm.model.scaffold1486.2 (78% identity with CYP76AK6 from *S. pomifera* and 73% identity with CYP76AK3 from *S. miltiorrhiza*), both belongs to the CYP76AK family following the classification of Chen *et al*.^55^ and are different enough to be consider two distinct CYP450s (Supplementary Fig. S4). Taking into account that their upregulation was higher in E2 than E5 samples and that CYP76AK3 has been related with the monoterpenes biosynthesis^55^ and CYP76AK6 with carnosic acid biosynthesis^57,58^, which share precursors with tanshinones biosynthesis pathway, could suggest their contribution upstream. CYP81D3 and CYP98A72 were theoretically annotated as isoflavone 2-hydrolase-like and p-coumarate 3-hydroxylase, respectively; indicating their role in phenolic compounds synthesis.

Other oxygenases as 2OGDs has been reported to take part in tanshinone biosynthesis^33^. We found ten different proteins corresponding to potential 2OGDs (Supplementary Table S3). The protein evm.model.scaffold10983.1 showed the highest upregulation and the same abundance profile of tanshinone biosynthesis related enzymes, 5.9 and 8.0-fold compared to the control in E2 and E5 samples respectively. This protein presented 60% identity with 2OGD5, which is the protein identified by Xu and Song^33^ to be involved in the downstream biosynthesis of tanshinones following the hydroxylation of CYPs and the unique 2OGD significantly expressed in roots. However, we found also the protein k15043_g1_i1 upregulated in E2 and E5 samples approximately with the same value (8.2 and 7.3-fold compared to the control) and it presented 66% identity with 2OGD5. The proteins CL18507Contig1, e13998_g1_i2, CL5300Contig1 and CL5240Contig1 were significantly upregulated only in E2 samples, specially CL18507Contig1 with 12.7-fold compared to the control.

We analyzed the expression profile of members of the family berberine bridge enzyme (BBE)-like enzymes found in the present work, grouped into the “FAD-binding berberine” GO category. A total of 15 BBE-like enzymes were induced under tanshinones elicitation (Supplementary Table S4), 5 of which presented an abundance profile in E2 and E5 in line with the tanshinones production observed (Fig. 6b). Interestingly, the expression level of some of these enzymes was significantly higher than any other tanshinone-related proteins and with a remarkable difference between the two group of samples analyzed. For instance, i14488_g1_i1 was up-regulated by 9.8-fold compared to the control in E2 samples and 29.5-fold in E5 and k17860_g1_i1 induced 5.1 and 18.3-fold in E2 and E5, respectively. Despite of the fact that these proteins have been related to alkaloid and cannabinoid biosynthesis, currently it is known that BBE-like enzymes can catalyze a wide variety of reactions. Daniel, *et al*.^49^ observed that most BBE-like enzymes from plants are phylogenetically clustered with fungal carbohydrate oxidases and among them, some BBE-like enzymes have been adapted to catalyze more complex reactions like oxidative ring closure reactions that remind the tanshinones cyclic structure. Recently, a BBE-like enzyme from *Arabidopsis thaliana* (AtBBE) was found to be involved in the plant immune response by Rajniak and coworkers^59^ synthetizing a new group of metabolites strongly upregulated under osmotic stress and after the exposure to pathogens. In addition, the identification of an specific active site in BBE-like proteins only present in the Brassicaceae family by Daniel, *et al*.^60^ suggested that depending of the plant, the BBE-like enzymes members and roles can be many and very variable. In view of the large number of uncharacterized BBE-like enzymes found in this analysis and the current efforts to understand the biosynthesis of tanshinones, it will be interesting to look forward their structural characterization and the physiological functions that BBE-like enzymes are involved in. Being the best candidates i14488_g1_i1, k17860_g1_i1, CL21093Contig1, evm.model. scaffold6028.3 and i7082_g2_i1.

## Conclusions

In summary, we have successfully demonstrated that LC-MS/MS proteomic approach is a useful tool to study the biosynthetic pathway of active compounds in plants. The unification, translation and annotation of different draft genome and transcriptome databases of *S. miltiorrhiza*, some of them currently not available, permitted us to obtain a very complete proteome database useful to our analysis. The use of 1 g L^−1^ and 0.41 mM of YE and Ag^+^ respectively to tanshinones production elicitation in *S. miltiorrhiza* hairy roots resulted to be highly efficient, reaching a total tanshinones concentration of 22 mg g^−1^ of DW, the highest reported to date. Despite the basic mechanism of tanshinone biosynthesis has not yet been fully elucidated, the proteins included in this pathway in particular CMK, HDS, AACT, GGPPS, CPS and KSL were highly induced at high tanshinone concentration, as well as the cytochromes P450 CYP76AH1, CYP76AH3 and CYP76AK1. New cytochromes P450 were identified as possible candidates for tanshinone biosynthesis based on their abundance profiles. Members of the BBE-like enzymes family showed the highest induction at maximum tanshinones production. It would be interesting to know if they are part of tanshinones biosynthetic pathway. In this context, Knock-out experiments are currently in progress. The elucidation of biosynthetic pathway will help to directly and efficiently produce active compounds of *S. miltiorrhiza* in hairy roots.

## Methods

### Plant Materials

*S. miltiorrhiza* hairy roots were supplied by Green2Chem company (Ghislenghien, Belgium). The roots were obtained by *Rhizobium rhizogenes* ATCC15834 infection, and the clone was selected based on their fast growth. They were cultivated in Gamborg’s B5 liquid medium^61^ supplemented with 0.5 g L^−1^ of activated carbon in 500-mL Erlenmeyer flasks on an orbital shaker at 70 rpm (Circular Motion Shaker, 3500) and 22 °C kept in the dark. The hairy roots were subcultured at 4-weeks intervals.

### Tanshinone elicitation

The elicitation consisted in the application of a biotic elicitor 1 g L^−1^ yeast extract (VWR-84601) and an abiotic elicitor 0.41 mM AgNO_3_ (Sigma-85228), individually or simultaneously. The YE was prepared in water at 0.15 g mL^−1^ concentration and sterilized by autoclaving and AgNO_3_ was supplied from aqueous solution 41 mM sterilized by 0.2 µm filtration. The elicitation of tanshinone production was started 15 days after inoculating 20 g fresh weight of hairy roots in 300 mL of medium. The elicitors were added to the existing medium, which was kept during all elicitation period. The hairy roots were harvested after 2, 4, 5 and 7 weeks post elicitation. Three individual flasks were collected in each time point. Fresh weight was estimated after blotting with absorbent paper. The fresh roots obtained were stored at −20 °C until lyophilization and further use.

### HPLC analysis

Compound extraction and analysis were performed as per the methods described by Xing *et al*.^22^ with minor modifications. The lyophilized hairy roots were grounded to powdered with a mortar and a pestle. The sample powder (50 mg) was extracted three times with 70% methanol (2 mL) under ultrasonication for 15 min and centrifugation at 14,000 x g for 5 min. The supernatant collected was filtered through a 0.45-mm Millipore filter and used for HPLC analysis. HPLC analysis was performed using an Alliance System (Waters, Milford, MA, USA) equipped with a binary pump, an automatic sample injector and a Waters 2996 photodiode array detector (PDA). An Ace 5 C18 column (4.6 mm × 250 mm, 5 mm particle size) was used at 50 °C. Empower Pro software was used for data acquisition and processing. The sample injection volume was set at 20 µL and the detection wavelength was 245 nm. Separation was achieved by elution using a linear gradient with solvent A (acetonitrile: MEOH 2:1) and solvent B (0.01% phosphoric acid solution). The gradient was as follows: t = 0 min, 70% A; t = 12.5 min, 85% A; t = 15 min, 85% A; t = 16 min, 70% A; t = 20 min, 70% A. The flow rate was set at 5 mL·min^−1^. Standards of metabolite compounds were purchased from Sigma Aldrich, except for the peak identify as 1, 2, 15, 16-tetrahydrotanshinone in which we used cryptotanshinone area as reference.

### Protein extraction and quantification

Total root protein was precipitated from 50 mg of lyophilized and grounded hairy roots with 10% (w/v) trichloroacetic acid (TCA) and 0.07% (v/v) 2-mercaptoethanol in acetone at −20 °C overnight. After centrifugation at 16,000 × g at 4 °C for 15 min, the pellets were washed 3 times with 0.07% (v/v) 2-mercaptoethanol in cold 80% acetone, dried in speed-vac for 10 minutes and kept at −20 °C until extraction^62^. The samples were solubilized in 1 mL resuspension buffer (6 M Guanidine-HCl, 5 mM DTE and 50 mM Tris-HCl pH 7.5) and sonication^63^. After centrifugation at 16,000 × g at 4 °C for 15 min the supernatant was recovered and quantified with Non-interfering agents kit (G-Biosciences 786-005) following the manufacturer’s instructions.

### Protein trypsin digestion

Protein (50 µg) was submitted to label-free differential proteomic analysis. Proteins were reduced adding 10 mM (W/V) dithioerythritol (DTE) in 25 mM NH_4_HCO_3_ for 20 min at 56 °C and alkylated by 25 mM (w/v) iodoacetamide in 25 mM NH_4_HCO_3_ for 30 min in darkness at room temperature. Proteins were recovered by acetone precipitation and dissolved in 20 µL 25 mM (w/v) NH_4_HCO_3_ (pH 8.5) containing 2 µg trypsin (Promega V51 11) and incubated at 37 °C overnight. Formic acid (0.2%, v/v, final) was used to stop trypsinization process^64^.

### LC-MS/MS analysis

Peptides were separated on a reverse-phase column (length 25 cm, diameter 75 µm, flowrate 300 nL min^−1^; PepMap C18; Dionex) equilibrated at 4% (v/v) ACN and submitted to a linear ACN gradient (4 to 35%, v/v) for 120 min. The column was previously equilibrated with 4% ACN for 20 min. The peptide elution was followed by a wash step performed with 90% (v/v) ACN for 10 min. MS analysis was performed with an AB Sciex TripleTOF 5600 System. Peptide mass spectra were acquired in data-dependent acquisition spectra under high sensitivity (HS) mode (MS: m/z 400–1500 and accumulation time 0.5 s; MS/MS: 50/cycle, m/z 100–1800 and accumulation time 0.05 s) leading to a duty cycle time of 3 s. The ion spray voltage was 2.3 kV, and nitrogen was used as a curtain gas. The TOF analyser was automatically calibrated with the beta-galactosidase tryptic digest every 10 h, allowing for the maintenance of a mean mass error below 10 ppm. across all injections^64^.

### Proteomic Database Search

Protein Pilot 4.0 (AB Sciex Inc., USA) was used to identify proteins using basic parameters and “thorough”^62,64^ analysis effort against *S. miltiorrhiza* database built mixing protein sequences obtained from genome^1^, transcriptome^4^ and National Center for Biotechnology Information (https://www.ncbi.nlm.nih.gov). After removing the redundant sequences using H-CD-HIT (Huang *et al*.^65^; http://weizhongli-lab.org/cdhit_suite/cgi-bin/index.cgi) by clustering at 95% identity, the sequences obtained were classified into functional plant categories using the software Mercator (Lohse *et al*.^66^; http://plabipd.de/portal/mercator-sequence-annotation). Mercator uses MapMan categories, which categorize proteins in metabolic pathways and enzyme functions. For the analysis three sequence classifications were performed: Blast searches against Arabidopsis TAIR10, TIGR5 rice proteins, plant proteins from swiss-prot and UniRef90, RPS-Blast searches against cdd and KOG and InterPro scan. A false discovery rate (FDR) analysis was accomplished by using the integrated tools in ProteinPilot™ software 4.5 with a confidence level of 99%.

### Protein quantification and Statistical Analysis

PeakView® software 2.1 (Absciex, USA) was used for XIC calculation for top 5 peptides of all proteins identified, considering an FDR below 1% (as determined by Protein Pilot)^67^. Markerview™ 1.2.1 was used for statistical treatment of the data^64,67^. Area under the XIC curves of peptides were individually normalized based on a summed area of all peptides for each sample. Three biological replicates were considered for each condition and each elicitation treatment was compared with its respective control sample. Differentially expressed proteins were required to fulfil 3 conditions for further consideration: (1) Quantification with at least 2 unique peptides; (2) the P-value lower than 0.05; and (3) fold changes higher than 1.5-fold or lower than 0.6-fold. All identified proteins were classified as it was mentioned above. The obtained MapMan bin-codes were sorted manually into 11 gene ontology classes as shown in Supplementary Table S1 and every protein was assigned a single bin-code.

## Supplementary information

Supplementary dataset

Supplementary materials
