# Supplementary material for: Proteomic analysis reveals novel insights into tanshinones biosynthesis in Salvia miltiorrhiza hairy roots
Source: Sci Rep. 2019 Apr 8;9:5768. doi: 10.1038/s41598-019-42164-3 (PMC6453882; doi:10.1038/s41598-019-42164-3)
Supplement: Supplementary file 2 — Supplementary materials [file 41598_2019_42164_MOESM2_ESM.docx]

**Proteomic analysis reveals novel insights into tanshinones biosynthesis in *Salvia miltiorrhiza* hairy roots.**

**Angela Contreras**^1^**, Baptiste Leroy**^1^**, Pierre-Antoine Mariage**^2^**, and Ruddy Wattiez**^1^*

| **Nº** | **Go category** | **Bin-codes description** | |
| --- | --- | --- | --- |
| 1 | Metabolism | Hormone metabolism | hormone metabolism.abscisic acid  hormone metabolism.auxin  hormone metabolism.brassinosteroid  hormone metabolism.cytokinin  hormone metabolism.ethylene  hormone metabolism.gibberelin  hormone metabolism.jasmonate  hormone metabolism.salicylic acid |
|  |  | Secondary metabolism | co-factor and vitamine metabolism  lipid metabolism.FA synthesis and FA elongation  lipid metabolism.glyceral metabolism  lipid metabolism.lipid degradation  lipid metabolism.lipid transfer proteins  misc.alcohol dehydrogenases  misc.cytochrome P450  misc.nitrilases, *nitrile lyases, FAD-binding berberine enzymes, reticuline oxidases, troponine reductases  secondary metabolim  secondary metabolism.flavonoids  secondary metabolism.isoprenoids.mevalonate pathway  secondary metabolism.isoprenoids.non-mevalonate pathway  secondary metabolism.N misc.alkaloid-like  secondary metabolism.phenylpropanoids  secondary metabolism.phenylpropanoids.lignin biosynthesis  secondary metabolism.simple phenols  secondary metabolism.sulfur-containing.glucosinolates |
|  |  | Carbohydrate metabolism | fermentation  gluconeogenesis / glyoxylate cycle  glycolysis.cytosolic branch  glycolysis.plastid branch  major CHO metabolism  misc.beta 1,3 glucan hydrolases  misc.gluco-, galacto- and mannosidases  OPP.oxidative PP |
|  |  | Other metabolism | amino acid metabolism  amino acid metabolism.degradation  amino acid metabolism.misc  amino acid metabolism.synthesis  biodegradation of Xenobiotics  C1-metabolism  misc.UDP glucosyl and glucoronyl transferases  N-metabolism.ammonia metabolism  N-metabolism.N-degradation  nucleotide metabolism  polyamine metabolism.synthesis  tetrapyrrole synthesis  xenobiotic metabolism |
|  |  | TCA cycle | TCA / org transformation |
| 2 | Stress |  | cell.cell death.plants  metal handling  stress.abiotic  stress.biotic  stress.biotic.PR-proteins |
| 3 | Redox |  | misc.glutathione S transferases  misc.oxidases - copper, flavone etc  misc.peroxidases  misc.short chain dehydrogenase/reductase (SDR)  OPP.electron transfer  redox.ascorbate and glutathione  redox.dismutases and catalases  redox.misc  redox.peroxiredoxin  redox.thioredoxin  S-assimilation.sulfite redox |
| 4 | Signalling |  | signalling.calcium  signalling.14-3-3 proteins  signalling.G-proteins  signalling.in sugar and nutrient physiology  signalling.light.COP9 signalosome  signalling.misc  signalling.phosphinositides  signalling.receptor kinases |
| 5 | Transport |  | cell.vesicle transport  transport.ABC transporters and multidrug resistance systems  transport.ammonium  transport.calcium  transport.H+ transporting pyrophosphatase  transport.Major Intrinsic Proteins  transport.metabolite transporters at the envelope membrane  transport.p- and v-ATPases  transport.porins  transport.potassium  transport.sugars  transport.unspecified cations |
| 6 | Protein |  | protein.aa activation  protein.degradation  protein.folding  protein.postranslational modification  protein.synthesis |

**Supplementary Table S1:** List of gene ontology terms categories grouped by the corresponding bin-codes.

| **Nº** | **Go category** | **Bin-codes description** | |
| --- | --- | --- | --- |
| 7 | Development |  | development.late embryogenesis abundant  development.storage proteins  development.unspecified  gene repression, organogenesis and meristem development |
| 8 | Cell wall |  | cell wall.cell wall proteins  cell wall.degradation  cell wall.modification  cell wall.pectin*esterases  cell wall.precursor synthesis |
| 9 | DNA/RNA |  | DNA.repair  DNA.synthesis/chromatin structure  DNA.unspecified  nucleotide metabolism.synthesis  RNA.processing  RNA.regulation of transcription  RNA.RNA binding  RNA.transcription |
| 10 | Others |  | cell.cycle  cell.division  cell.organisation  cell.organisation.cytoskeleton  cell.vesicle transport  fermentation.aldehyde dehydrogenase  misc.acid and other phosphatases  misc.acyl transferases  misc.aminotransferases.aminotransferase class IV family protein  misc.dynamin  misc.GDSL-motif lipase  misc.invertase/pectin methylesterase inhibitor family protein  misc.misc2  misc.O-methyl transferases  misc.plastocyanin-like  misc.protease inhibitor/seed storage/lipid transfer protein (LTP) family protein  mitochondrial electron transport / ATP synthesis  PS.calvin cycle  PS.lightreaction  PS.photorespiration |
| 11 | Not assigned |  | not assigned.no ontology  not assigned.unknown |

**Supplementary Table S2.** List of metabolism proteins increasing significantly their abundance after YE + Ag^+^ treatment.

| **Sequence ID** | **Description** | **GO category** | **pep** | **FC** | **p-value** |  |  |
| --- | --- | --- | --- | --- | --- | --- | --- |
|  |  |  |  | **E2** | |  | |
| CL11482Contig1 | Bifunctional epoxide hydrolase 2-like | Other metabolism.xenobiotic metabolism | 2 | **20.58** | 0.044 |  |  |
| evm.model.scaffold348.3 | Anthocyanidin 3-O-glucosyltransferase | Flavonoids metabolism | 4 | **6.09** | 0.008 |  |  |
| CL4072Contig1 | Acyl-coenzyme a oxidase peroxisomal-like | Lipid metabolism.beta-oxidation.acyl coa DH | 4 | **5.15** | 0.033 |  |  |
| evm.model.scaffold4068.1 | Ent-kaurene synthase B | Isoprenoids metabolism | 6 | **4.49** | 0.005 |  |  |
| evm.model.C221605.6 | Zeatin O-xylosyltransferase | Phenylpropanoids.lignin biosynthesis | 7 | **4.22** | 0.011 |  |  |
| evm.model.scaffold1486.2 | CYP76AK6 [sp] | Cytochrome P450 | 2 | **3.30** | 0.028 |  |  |
| evm.model.scaffold3630.13 | UDP-glucosyl transferase 85A2 (UGT85A2) | Hormone metabolism.cytokinin.synthesis-degradation | 3 | **3.29** | 0.009 |  |  |
| evm.model.scaffold581.16 | Phosphoglucosamine mutase family protein | Carbohydrate metabolism | 4 | **3.19** | 0.001 |  |  |
| CL8387Contig1 | Quercetin 3-o-glucoside-6''-o-malonyltransferase | Flavonoids.anthocyanins | 9 | **3.19** | 0.010 |  |  |
| CL2846Contig1 | 1-deoxy-d-xylulose-5-phosphate synthase | Isoprenoids.non-mevalonate pathway | 11 | **3.17** | 0.018 |  |  |
| CL4482Contig2 | FAD-binding Berberine family protein | FAD-binding berberine | 29 | **3.10** | 0.001 |  |  |
| CL4770Contig1 | Bifunctional methylthioribulose-1-phosphate dehydratase | Carbohydrate metabolism | 6 | **3.01** | 0.014 |  |  |
| e13998_g1_i2 | 2-oxoglutarate (2OG) and Fe(II)-dependent oxygenase | Flavonoids.flavonols | 4 | **2.84** | 0.047 |  |  |
| CL859Contig2 | UDP-glycosyltransferase 88a1-like isoform 1 | Other metabolism.UDP glucosyl and glucoronyl transferases | 7 | **2.76** | 0.023 |  |  |
| evm.model.scaffold61.7 | FAD-dependent oxidoreductase family protein | Other metabolism.amino acid metabolism.synthesis | 6 | **2.60** | 0.015 |  |  |
| evm.model.C220153.7 | Aspartate aminotransferase | Other metabolism.amino acid metabolism | 4 | **2.48** | 0.001 |  |  |
| CL64Contig10 | UDP-glycosyltransferase 74e2-like | Sulfur-containing.glucosinolates | 9 | **2.45** | 0.009 |  |  |
| CL5300Contig1 | 2-oxoglutarate (2OG) and Fe(II)-dependent oxygenase | Secondary metabolism.flavonoids.flavonols | 9 | **2.43** | 0.004 |  |  |
| CL7039Contig1 | UDP-glycosyltransferase 85a2-like | Hormone metabolism.cytokinin.synthesis-degradation | 11 | **2.43** | 0.024 |  |  |
| evm.model.scaffold9917.4 | Phenylcoumaran benzylic ether reductase (PCBER)-Like | Flavonoids.isoflavones | 18 | **2.10** | 0.030 |  |  |
| CL4930Contig1 | Phenylcoumaran benzylic ether reductase (PCBER) | Flavonoids.isoflavones | 19 | **2.10** | 0.031 |  |  |
| CL481Contig4 | Alcohol dehydrogenase-like 7-like | Other metabolism.alcohol dehydrogenases | 2 | **2.08** | 0.038 |  |  |
| k20098_g1_i1 | Bifunctional nitrilase nitrile hydratase nit4b-like | Sulfur-containing.glucosinolates | 25 | **1.96** | 0.016 |  |  |
| CL20639Contig1 | FAD-binding Berberine family protein | FAD-binding berberine | 22 | **1.95** | 0.020 |  |  |
| CL6567Contig1 | FAD-binding Berberine family protein | FAD-binding berberine | 27 | **1.94** | 0.002 |  |  |
| CL5089Contig1 | NAD(P)-linked oxidoreductase | Hormone metabolism.auxin | 6 | **1.88** | 0.026 |  |  |
| evm.model.scaffold3987.37.2 | Citrate synthase 5 | TCA / org transformation.TCA.CS | 38 | **1.86** | 0.014 |  |  |
| CL9603Contig1 | Bifunctional nitrilase nitrile hydratase nit4b-like | Sulfur-containing.glucosinolates | 13 | **1.85** | 0.004 |  |  |
| a8224_g1_i1 | Sucrose synthase | Carbohydrate metabolism | 100 | **1.84** | 0.023 |  |  |
| k18756_g1_i1 | Pyruvate cytosolic isozyme-like | Carbohydrate metabolism | 29 | **1.72** | 0.008 |  |  |
| CL240Contig4 | Phosphomevalonate kinase-like | Isoprenoids.mevalonate pathway | 9 | **1.65** | 0.010 |  |  |
| CL436Contig4 | Phenylalanine ammonia-lyase | Phenylpropanoids.lignin biosynthesis | 24 | **1.61** | 0.031 |  |  |
| CL2061Contig1 | Glyoxysomal fatty acid beta-oxidation multifunctional | Lipid metabolism.lipid degradation | 29 | **1.58** | 0.011 |  |  |
| evm.model.C220963.4 | 6-phosphogluconolactonase 2 | Carbohydrate metabolism | 10 | **1.53** | 0.018 |  |  |
| CL5871Contig2 | 2-C-methyl-D-erythritol 4-phosphate cytidylyltransferase | Isoprenoids.non-mevalonate pathway | 7 | **1.52** | 0.030 |  |  |
| **Sequence ID** | **Description** | **GO category** | **pep** | **FC** | **p-value** | **FC** | **p-value** |
|  |  |  |  | **E2** | | **E5** | |
| i14488_g1_i1 | FAD-binding Berberine family protein | FAD-binding berberine | 5 | **9.87** | 0.010 | **29.50** | 0.001 |
| CL6457Contig1 | Zeatin o-glucosyltransferase-like | Phenylpropanoids.lignin biosynthesis | 14 | **7.34** | 0.002 | **27.48** | 0.000 |
| CL415Contig3 | Geranylgeranyl diphosphate synthase | Isoprenoids.non-mevalonate pathway | 5 | **4.77** | 0.001 | **19.17** | 0.000 |
| k17860_g1_i1 | FAD-binding Berberine family protein | FAD-binding berberine | 6 | **5.18** | 0.012 | **18.37** | 0.000 |
| CL21093Contig1 | FAD-binding Berberine family protein | FAD-binding berberine | 14 | **2.46** | 0.010 | **18.01** | 0.000 |
| k16834_g1_i1 | FAD-binding Berberine family protein | N misc.alkaloid-like | 42 | **5.92** | 0.002 | **14.39** | 0.004 |
| evm.model.scaffold6028.3 | FAD-binding Berberine family protein | FAD-binding berberine | 41 | **5.77** | 0.002 | **14.36** | 0.003 |
| CL15092Contig1 | Geraniol 8-hydroxylase-like CYP76AK1 | Cytochrome P450 99% AMB36497.1-CYP76AK1 | 18 | **6.21** | 0.000 | **12.91** | 0.001 |
| CL10657Contig1 | S-adenosyl-l-methionine-dependent methyltransferases | Hormone metabolism.salicylic acid.synthesis-degradation | 6 | **4.75** | 0.005 | **12.41** | 0.008 |
| i3423_g1_i1 | FAD-binding Berberine family protein | N misc.alkaloid-like | 2 | **9.65** | 0.003 | **9.51** | 0.020 |
| evm.model.scaffold10983.1 | 2-oxoglutarate-dependent dioxygenase | Hormone metabolism.ethylene.synthesis-degradation | 7 | **5.94** | 0.003 | **8.03** | 0.002 |
| evm.model.scaffold4851.6 | FAD-binding Berberine family protein | N misc.alkaloid-like | 28 | **7.20** | 0.000 | **7.91** | 0.001 |
| i7082_g2_i1 | FAD-binding Berberine family protein | FAD-binding berberine | 18 | **3.44** | 0.001 | **7.58** | 0.000 |
| k19819_g1_i5 | Cytochrome p450 71d11-like | Sulfur-containing.glucosinolates | 4 | **4.38** | 0.000 | **7.45** | 0.000 |
| k15043_g1_i1 | 2-oxoglutarate-dependent dioxygenase | Sulfur-containing.glucosinolates.synthesis.aliphatic | 8 | **8.27** | 0.013 | **7.36** | 0.001 |
| CL1147Contig3 | Laccase-14-like | Other metabolism.simple phenols | 18 | **2.53** | 0.010 | **5.94** | 0.000 |
| CL2946Contig1 | 65%- cyp71d411 | Cytochrome P450 | 23 | **3.82** | 0.002 | **5.43** | 0.000 |
| evm.model.scaffold10190.3 | Aldehyde dehydrogenase | Carbohydrate metabolism | 20 | **2.75** | 0.008 | **4.48** | 0.000 |
| CL6853Contig2 | Geraniol 8-hydroxylase-like/ CYP76AK3 | Cytochrome P450 | 15 | **7.56** | 0.000 | **4.43** | 0.013 |
| CL1201Contig1 | 4-hydroxy-3-methylbut-2-en-1-yl diphosphate synthase | Isoprenoids.non-mevalonate pathway.HDS | 49 | **3.29** | 0.000 | **4.40** | 0.001 |
| CL2989Contig1 | Amb36496.1-cyp76ah3 | Cytochrome P450 | 26 | **4.13** | 0.002 | **4.34** | 0.002 |
| CL4821Contig1 | FAD-binding Berberine family protein | N misc.alkaloid-like | 32 | **3.49** | 0.001 | **4.06** | 0.038 |
| CL23195Contig1 | Geranyl diphosphate synthase small subunit type | Isoprenoids.non-mevalonate pathway | 5 | **2.29** | 0.036 | **3.77** | 0.002 |
| evm.model.scaffold10739.4 | FAD-binding Berberine family protein | N misc.alkaloid-like | 54 | **1.95** | 0.001 | **3.71** | 0.007 |
| evm.model.scaffold8828.2 | NAD(P)-linked oxidoreductase superfamily protein | Hormone metabolism.auxin. | 17 | **4.08** | 0.001 | **3.64** | 0.000 |
| CL3294Contig1 | Phenylcoumaran benzylic ether reductase | Flavonoids.isoflavones.isoflavone reductase | 51 | **2.93** | 0.001 | **3.59** | 0.000 |
| CL2705Contig1 | Vacuolar invertase | Carbohydrate metabolism | 24 | **3.16** | 0.011 | **3.43** | 0.008 |
| ACR57218.1 | Cytochrome P450-CYP71D411 | Flavonoids.dihydroflavonols.flavonoid 3''-monooxygenase | 36 | **3.84** | 0.000 | **3.41** | 0.000 |
| evm.model.scaffold1391.1 | FAD-binding Berberine family protein | FAD-binding berberine | 41 | **2.82** | 0.002 | **3.38** | 0.011 |
| CL301Contig9 | Probable aldo-keto reductase 2-like | Hormone metabolism.auxin | 35 | **3.98** | 0.000 | **3.28** | 0.001 |
| CL1998Contig1 | Glucose-6-phosphate dehydrogenase | Carbohydrate metabolism | 32 | **2.35** | 0.001 | **3.16** | 0.000 |
| CL20Contig23 | Polyphenol oxidase | Other metabolism | 6 | **3.13** | 0.001 | **3.05** | 0.000 |
| CL4482Contig1 | FAD-binding Berberine family protein | FAD-binding berberine | 30 | **3.14** | 0.003 | **3.03** | 0.034 |
| CL445Contig1 | Methylenetetrahydrofolate reductase 2-like | Other metabolism | 34 | **1.98** | 0.044 | **2.95** | 0.000 |
| evm.model.scaffold10690.3 | Copalyl diphosphate synthase | Hormone metabolism.gibberelin.synthesis-degradation | 28 | **2.16** | 0.006 | **2.91** | 0.002 |
| CL253Contig9 | Secologanin synthase-like 90% AJD25170.1-CYP72A329 [SM] | Misc.cytochrome P450. Monoterpenes | 21 | **2.30** | 0.011 | **2.85** | 0.015 |
| CL1935Contig4 | Quercetin 3-o-glucoside-6''-o-malonyltransferase | Flavonoids.anthocyanins | 15 | **3.18** | 0.015 | **2.73** | 0.001 |
| CL19981Contig1 | Polyphenol oxidase | Other metabolism | 43 | **4.02** | 0.000 | **2.71** | 0.001 |
| CL7305Contig1 | 1-deoxy-d-xylulose-5-phosphate reductoisomerase | Isoprenoids.non-mevalonate pathway.DXR | 21 | **2.62** | 0.029 | **2.67** | 0.050 |
| CL3840Contig2 | Salicylic acid-binding protein 2-like | FAD-binding berberine | 12 | **2.18** | 0.002 | **2.67** | 0.001 |
| evm.model.scaffold8520.3 | Acetone-cyanohydrin lyase | FAD-binding berberine | 8 | **2.39** | 0.002 | **2.41** | 0.000 |
| evm.model.scaffold4779.9 | NAD(P)-linked oxidoreductase superfamily protein | Hormone metabolism.auxin | 62 | **3.59** | 0.000 | **2.40** | 0.002 |
| CL1722Contig1 | Beta-amyrin synthase | Lipid metabolism.exotics (steroids, squalene etc) | 10 | **2.10** | 0.004 | **2.20** | 0.029 |
| CL9030Contig1 | Isoflavone 2 -hydroxylase-like | Cytochrome P450 | 17 | **3.18** | 0.012 | **2.12** | 0.026 |
| l1672_g1_i1 | AJD25181.1-ferruginol synthase | Cytochrome P450- CYP76AH1 | 25 | **2.51** | 0.003 | **2.11** | 0.016 |
| CL4324Contig1 | Cytochrome p450 reductase | Cytochrome P450 | 13 | **1.98** | 0.040 | **2.10** | 0.014 |
| CL7002Contig1 | Quercetin 3-o-glucoside-6''-o-malonyltransferase | Flavonoids.anthocyanins | 11 | **1.69** | 0.039 | **2.06** | 0.023 |
| CL803Contig2 | 3-ketoacyl-coa thiolase peroxisomal-like | Other metabolism.amino acid metabolism.degradation | 41 | **1.84** | 0.009 | **2.01** | 0.008 |
| CL3054Contig1 | Pyrroline-5-carboxylate synthetase | Other metabolism.amino acid metabolism | 21 | **2.32** | 0.027 | **1.96** | 0.000 |
| CL495Contig3 | Phospholipase d beta 1-like | Lipid metabolism.lipid degradation | 9 | **2.39** | 0.025 | **1.93** | 0.013 |
| CL803Contig3 | 3-ketoacyl-coa thiolase peroxisomal-like | Other metabolism.amino acid metabolism.degradation | 28 | **1.92** | 0.024 | **1.90** | 0.030 |
| CL655Contig3 | 4-hydroxy-3-methylbut-2-enyl diphosphate reductase | Isoprenoids.non-mevalonate pathway | 11 | **2.26** | 0.038 | **1.87** | 0.025 |
| CL7979Contig1 | 2-c-methyl-d-erythritol 2,4-cyclodiphosphate synthase | Isoprenoids.non-mevalonate pathway | 4 | **3.31** | 0.002 | **1.85** | 0.036 |
| CL5178Contig1 | Glutamate dehydrogenase 2-like | Other metabolism.N-metabolism.N-degradation | 42 | **2.50** | 0.005 | **1.83** | 0.000 |
| CL8505Contig1 | Citrate synthase peroxisomal-like | Carbohydrate metabolism | 5 | **1.62** | 0.008 | **1.77** | 0.001 |
| evm.model.scaffold1037.9 | Cinnamyl alcohol dehydrogenase homolog 3 | Phenylpropanoids.lignin biosynthesis | 21 | **1.62** | 0.041 | **1.71** | 0.006 |
| CL5985Contig1 | Dihydropyrimidine dehydrogenase | Other metabolism.nucleotide metabolism | 10 | **2.46** | 0.006 | **1.62** | 0.047 |
| CL11641Contig1 | Cinnamyl alcohol dehydrogenase | Phenylpropanoids.lignin biosynthesis | 13 | **1.52** | 0.040 | **1.58** | 0.011 |
| evm.model.scaffold3478.35 | Alanine aminotransferas | Other metabolism.amino acid metabolism.synthesis | 35 | **2.08** | 0.014 | **1.50** | 0.020 |
| **Sequence ID** | **Description** | **GO category** | **pep** | **FC** | **p-value** |  |  |
|  |  |  |  | **E5** | |  |  |
| CL1805Contig1 | Apoplastic invertase | Carbohydrate metabolism | 3 | **59.51** | 0.001 |  |  |
| evm.model.scaffold2965.1 | 4-(cytidine 5-phospho)-2-C-methyl-D-erithritol kinase | Isoprenoids.non-mevalonate pathway | 2 | **17.63** | 0.007 |  |  |
| CL12921Contig1 | Laccase-7- partial | Phenylpropanoids | 10 | **12.45** | 0.000 |  |  |
| a23791_g1_i1 | Cystathionine gamma synthase | Other metabolism.amino acid metabolism.synthesis | 2 | **5.98** | 0.015 |  |  |
| CL293Contig1 | Porphobilinogen chloroplastic-like | Other metabolism.tetrapyrrole synthesis | 2 | **5.92** | 0.030 |  |  |
| CL11768Contig1 | Geraniol dehydrogenase | Phenylpropanoids.lignin biosynthesis | 81 | **5.71** | 0.000 |  |  |
| CL15747Contig1 | Apoplastic invertase | Carbohydrate metabolism | 15 | **5.29** | 0.001 |  |  |
| k16786_g1_i1 | FAD-binding Berberine family protein | FAD-binding berberine | 10 | **4.78** | 0.002 |  |  |
| evm.model.scaffold4020.10 | Glutamate-ammonia ligases | Other metabolism.N-metabolism.ammonia metabolism | 5 | **4.47** | 0.009 |  |  |
| CL14095Contig1 | Copalyl diphosphate synthase | Hormone metabolism.gibberelin.synthesis-degradation | 33 | **3.97** | 0.001 |  |  |
| k2957_g1_i1 | Gibberellin-regulated protein 14-like | Hormone metabolism.gibberelin | 3 | **3.39** | 0.007 |  |  |
| evm.model.scaffold9725.2 | Long-chain acyl-coa synthetase | Lipid metabolism.FA synthesis and FA elongation | 8 | **3.36** | 0.001 |  |  |
| evm.model.scaffold1334.3 | Glucan endo-1,3-beta-glucosidase | Carbohydrate metabolism | 8 | **3.31** | 0.008 |  |  |
| CL9170Contig1 | Alpha-galactosidase 1 | Carbohydrate metabolism | 35 | **2.99** | 0.001 |  |  |
| CL5066Contig1 | Brassinosteroid insensitive 1-associated receptor kinase 1-like | Hormone metabolism.brassinosteroid.signal transduction | 6 | **2.76** | 0.018 |  |  |
| CL1819Contig2 | Probable carboxylesterase 15-like | Lipid metabolism.lipid degradation.lysophospholipases | 5 | **2.64** | 0.003 |  |  |
| CL15375Contig1 | Laccase-14-like | Other metabolism.simple phenols | 37 | **2.62** | 0.006 |  |  |
| CL3416Contig3 | Glucan endo-1,3-beta-glucosidase-like | Carbohydrate metabolism | 6 | **2.54** | 0.001 |  |  |
| CL2074Contig2 | FAD-binding Berberine family protein | FAD-binding berberine | 83 | **2.46** | 0.009 |  |  |
| CL11754Contig2 | FAD-binding Berberine family protein | FAD-binding berberine | 14 | **2.39** | 0.019 |  |  |
| CL4656Contig2 | UDP-glucosyl transferase 73B3 | Flavonoids.flavonols.flavonol 3-O-glycosyltransferase | 5 | **2.34** | 0.002 |  |  |
| CL9063Contig1 | Beta-1,3-glucanase | Carbohydrate metabolism | 12 | **2.32** | 0.034 |  |  |
| CL22581Contig1 | Hydroxycinnamoyl-coenzyme A | Phenylpropanoids | 2 | **2.16** | 0.043 |  |  |
| evm.model.scaffold11008.4 | Farnesyl diphosphate synthase 1 | Isoprenoids.mevalonate pathway | 17 | **2.02** | 0.010 |  |  |
| CL8413Contig1 (AT1G12010.1 2OGD) | 1-aminocyclopropane-1-carboxylate oxidase | Hormone metabolism.ethylene.synthesis-degradation | 4 | **1.99** | 0.046 |  |  |
| CL18325Contig1 | FAD-binding Berberine family protein | FAD-binding berberine | 9 | **1.91** | 0.041 |  |  |
| CL12951Contig1 | Cytochrome p450 - 77% AMZ03382.1-CYP71D379 | Cytochrome P450 | 5 | **1.88** | 0.041 |  |  |
| evm.model.scaffold8673.7 | NAD(P)-binding Rossmann-fold superfamily protein | Phenylpropanoids.lignin biosynthesis.CCR1 | 10 | **1.87** | 0.042 |  |  |
| evm.model.scaffold7861.2 | Probable mannitol dehydrogenase | Phenylpropanoids.lignin biosynthesis.CAD | 124 | **1.86** | 0.004 |  |  |
| CL5352Contig1 | Lipoxygenase | Hormone metabolism.jasmonate.synthesis-degradation | 44 | **1.83** | 0.001 |  |  |
| k4775_g1_i1 | Beta- insoluble isoenzyme cwinv1-like | Carbohydrate metabolism | 6 | **1.79** | 0.012 |  |  |
| CL2074Contig1 | FAD-binding Berberine family protein | FAD-binding berberine | 91 | **1.79** | 0.001 |  |  |
| CL11733Contig1 | Alcohol dehydrogenase-like 3-like | Other metabolism.alcohol dehydrogenases | 4 | **1.76** | 0.006 |  |  |
| CL1776Contig1 | Isopentenyl pyrophosphate isomerase | Isoprenoids.mevalonate pathway | 15 | **1.75** | 0.046 |  |  |
| AGW27206.1 | Phenylalanine ammonia-lyase 3 | Phenylpropanoids.lignin biosynthesis.PAL | 29 | **1.68** | 0.009 |  |  |
| k19656_g1_i1 | Polyphenol oxidase | Other metabolism | 70 | **1.66** | 0.001 |  |  |
| CL1193Contig3 | Betaine aldehyde dehydrogenase | Other metabolism.N misc.betaine | 27 | **1.65** | 0.025 |  |  |
| evm.model.scaffold379.3 | S-adenosylmethionine synthetase 2 | Other metabolism.amino acid metabolism.synthesis | 24 | **1.63** | 0.001 |  |  |
| CL8371Contig1 | Nucleoside diphosphate kinase chloroplastic mitochondrial-like | Other metabolism | 8 | **1.61** | 0.014 |  |  |
| CL1426Contig2 | Probable mannitol dehydrogenase-like | Phenylpropanoids.lignin biosynthesis | 146 | **1.56** | 0.021 |  |  |
| CL549Contig2 | Aconitate hydratase mitochondrial-like | TCA / org transformation.TCA | 71 | **1.50** | 0.001 |  |  |

**Supplementary Table S3.** List of 2OGDs proteins changing their abundance under YE + Ag^+^ treatment in *S. miltiorrhiza* hairy roots.

| **Sequence ID** | **Description** | **GO category** | **pep** | **FC** | **p-value** | **FC** | **p-value** |
| --- | --- | --- | --- | --- | --- | --- | --- |
|  |  |  |  | **E2** | | **E5** | |
| CL18507Contig1 | 2-oxoglutarate-dependent dioxygenase dao-like | Redox | 12 | **12.69** | 0.003 | **3.14** | 0.077 |
| e13998_g1_i2 | 2-oxoglutarate (2OG) and Fe(II)-dependent oxygenase | Secondary metabolism | 4 | **2.84** | 0.047 | **2.22** | 0.111 |
| CL5300Contig1 | 2-oxoglutarate (2OG) and Fe(II)-dependent oxygenase | Secondary metabolism | 9 | **2.43** | 0.004 | **0.74** | 0.135 |
| CL5240Contig1 | Oxoglutarate/iron-dependent oxygenase | Redox | 8 | **2.28** | 0.005 | **1.09** | 0.814 |
| evm.model.scaffold10983.1 | 2-oxoglutarate-dependent dioxygenase | Hormone metabolism | 7 | **5.94** | 0.003 | **8.03** | 0.002 |
| k15043_g1_i1 | 2-oxoglutarate-dependent dioxygenase | Secondary metabolism | 8 | **8.27** | 0.013 | **7.36** | 0.001 |
| CL34Contig1 | 2-oxoglutarate-dependent dioxygenase | Hormone metabolism | 7 | **0.17** | 0.006 | **0.10** | 0.007 |
| evm.model.scaffold775.3 | 2-oxoglutarate (2OG) and Fe(II)-dependent oxygenase | Hormone metabolism | 2 | **0.06** | 0.136 | **0.03** | 0.013 |
| CL8413Contig1 | 1-aminocyclopropane-1-carboxylate oxidase. (AT1G12010.1 2OGD) | Hormone metabolism | 4 | **0.89** | 0.790 | **1.99** | 0.046 |
| k20436_g2_i4 | 1-aminocyclopropane-1-carboxylate oxidase homolog 1-like | Hormone metabolism | 5 | **0.72** | 0.041 | **0.53** | 0.032 |
| k19027_g2_i1 | 1-aminocyclopropane-1-carboxylate oxidase 5-like (2OGD) | Hormone metabolism | 4 | **1.56** | 0.043 | **0.08** | 0.000 |

**Supplementary Table S4.** List of FAD-binding berberine proteins changing their abundance under YE + Ag^+^ treatment in *S. miltiorrhiza* hairy roots.

| **Sequence ID** | **Description** | **pep** | **FC** | **p-value** | **FC** | **p-value** |
| --- | --- | --- | --- | --- | --- | --- |
|  |  |  | **E2** | | **E5** | |
| CL4482Contig2 | FAD-binding Berberine family protein | 29 | **3.10** | 0.001 | **3.03** | 0.058 |
| CL20639Contig1 | FAD-binding Berberine family protein | 22 | **1.95** | 0.02 | **2.45** | 0.097 |
| CL6567Contig1 | FAD-binding Berberine family protein | 27 | **1.94** | 0.002 | **2.65** | 0.118 |
| i14488_g1_i1 | FAD-binding Berberine family protein | 5 | **9.87** | 0.01 | **29.50** | 0.001 |
| k17860_g1_i1 | FAD-binding Berberine family protein | 6 | **5.18** | 0.012 | **18.37** | 0.000 |
| CL21093Contig1 | FAD-binding Berberine family protein | 14 | **2.46** | 0.01 | **18.01** | 0.000 |
| evm.model.scaffold6028.3 | FAD-binding Berberine family protein | 41 | **5.77** | 0.002 | **14.36** | 0.003 |
| i7082_g2_i1 | FAD-binding Berberine family protein | 18 | **3.44** | 0.001 | **7.58** | 0.000 |
| evm.model.scaffold1391.1 | FAD-binding Berberine family protein | 41 | **2.82** | 0.002 | **3.38** | 0.011 |
| CL4482Contig1 | FAD-binding Berberine family protein | 30 | **3.14** | 0.003 | **3.03** | 0.034 |
| k16786_g1_i1 | FAD-binding Berberine family protein | 10 | **5.66** | 0.110 | **4.78** | 0.002 |
| CL2074Contig2 | FAD-binding Berberine family protein | 83 | **1.42** | 0.067 | **2.46** | 0.009 |
| CL11754Contig2 | FAD-binding Berberine family protein | 14 | **0.99** | 0.930 | **2.39** | 0.019 |
| CL18325Contig1 | FAD-binding Berberine family protein | 9 | **0.86** | 0.030 | **1.91** | 0.041 |
| CL2074Contig1 | FAD-binding Berberine family protein | 91 | **1.11** | 0.439 | **1.79** | 0.001 |

**Supplementary Figure S1.** Chromatogram of different tanshinones analyzed in control (C) and elicited (E) samples at 2, 5 and 7 weeks. SaA, salvianolic acids; DHt, dihydrotanshinone; THt, tetrahydrotanshinone; TI, tanshinone I; CTt, cryptotanshinone; TII, tanshinone II.


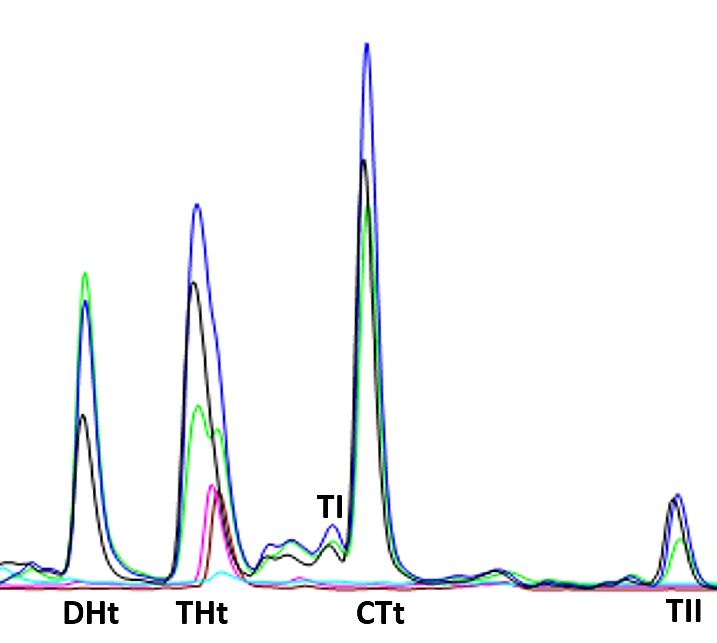

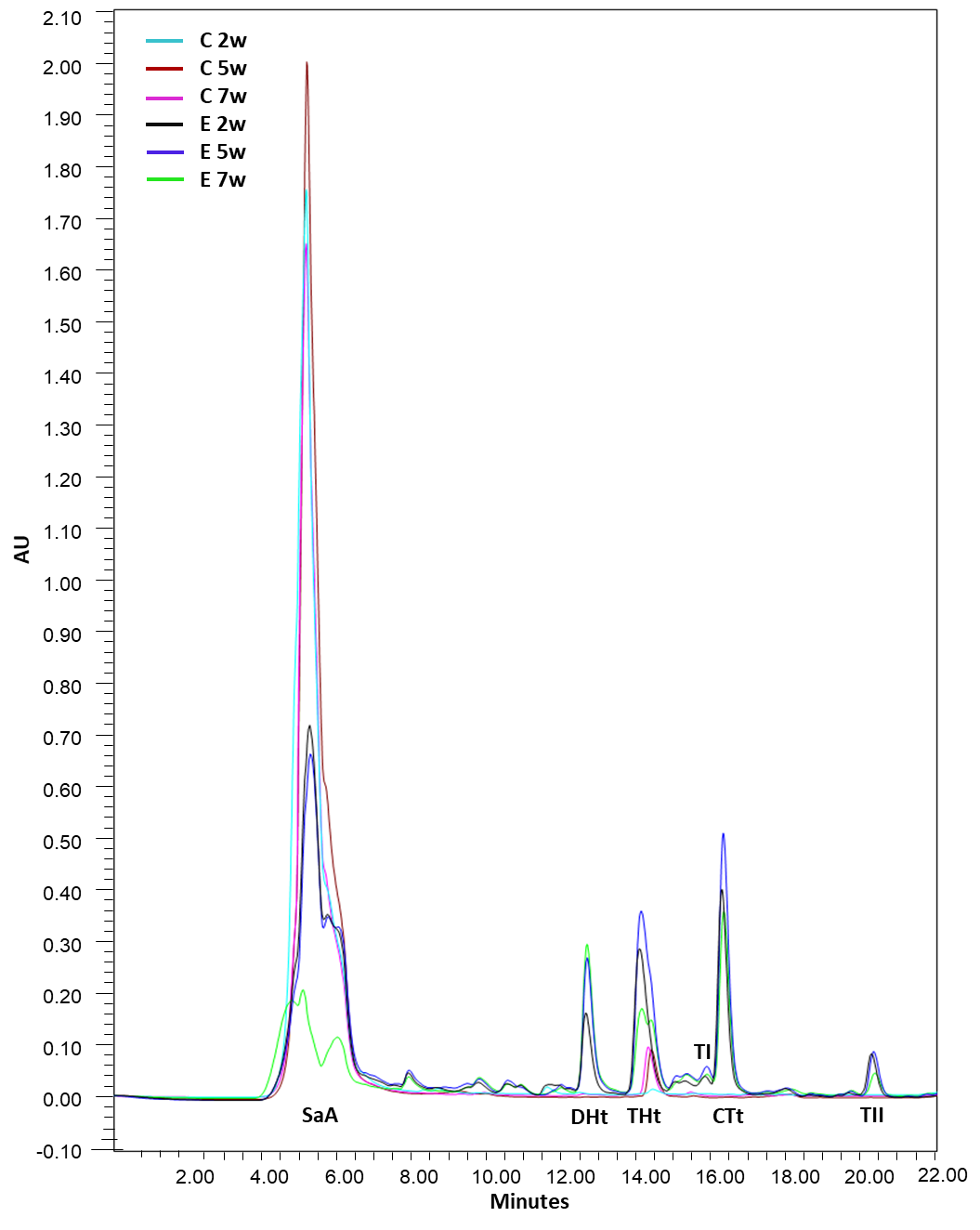


Supplementary Figure S2. Mercator annotation of Salvia miltiorrhiza proteomic database assigned to 35 different GO categories.

**
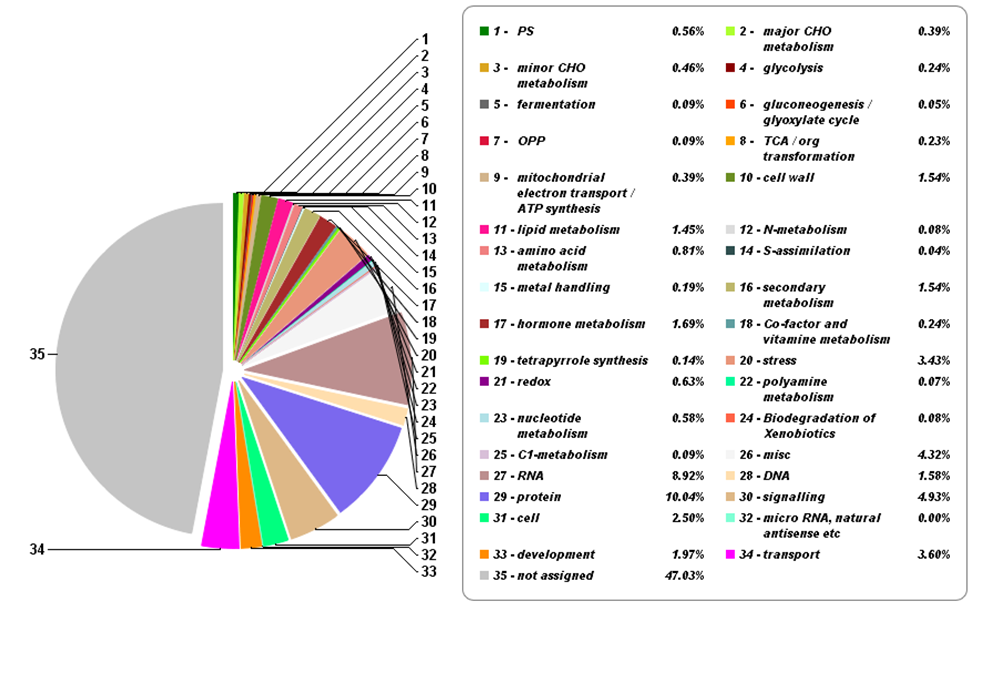
**

Supplementary Figure S3. Alignment of sequences CYP71D411-like.

>k19819_g1_i5

MVTALVLDSLRLGSGFMIADLYPSIKLLPLITGAQFRIQRIHHRLDRLFDTIIKQHKSAAKIDDDDDSIEDLVDVLLKVQKDGSLTTETIKAVLLNMFTAGTDTSTTAIEWAMSEMIKNPSKLNKAQEEVRKVFDNKGYVEEDKFDELKYLKLIIKETLRLHPPVPLLIPRMSSQRCEIDGYEIPAKTTVIVNAWALGRDPEYWKDPEKFIPERFEESSIDFKGNNLEYIPFGAGRRMCPGMTFGLANVELPLAMLLYHFDWKMPKGIKNEDLDMTEAFGVTVKRKHHLHLIPIVKRPLPAR

>CL2946Contig1 65%CYP71D411

MEFNISSTLIALLPFLLFLYTFLKYLQISKTANTYSHIPGPKTLPLIGNLHLMLSSPMPNYVFRDLAAKHGPLMHLQLGELPILIVSSIDLANQVVKTHDLIFANRPPGLAPETVAYNRTNIVFSPYGDHWRQLRRICTQELLNARRVQSFRPIREEENMNLCRWIASCEGSPANLSDKLYLTSYDVITRASVKAKTKEREAVVAVIIESLKVGAGFLLSDFYPSINLLPVITGALFRIQRVHRKFDKLLDGIIKQHRARAAANTTTNGQIVDDDDKYEDFVDVLLKFERDGSLTTANIKAVLLDMFLGGTDTSANTVEWAMSELIKNPSELSKAQEEVRRVFDDKGYVDEDKFDELKYLKLIIKETMRLHPALPLLLPRMSSQRCEINGYEIPAGTRVIVNAWALGRDPKYWKDAEKFIPERFEESSHDFHATNLEYIPFGAGRRICPGISFGIANVQLPLSMLLYHFDWKMPNGIKGEDLDMSEAFGITVNRNHHLHLVPTVKRPLSAAA

CLUSTAL O (1.2.4) multiple sequence alignment

k19819_g1_i5 ------------------------------------------------------------

CL2946Contig1 MEFNISSTLIALLPFLLFLYTFLKYLQISKTANTYSHIPGPKTLPLIGNLHLMLSSPMPN

k19819_g1_i5 ------------------------------------------------------------

CL2946Contig1 YVFRDLAAKHGPLMHLQLGELPILIVSSIDLANQVVKTHDLIFANRPPGLAPETVAYNRT

k19819_g1_i5 ------------------------------------------------------------

CL2946Contig1 NIVFSPYGDHWRQLRRICTQELLNARRVQSFRPIREEENMNLCRWIASCEGSPANLSDKL

k19819_g1_i5 ---------------------MVTALVLDSLRLGSGFMIADLYPSIKLLPLITGAQFRIQ

CL2946Contig1 YLTSYDVITRASVKAKTKEREAVVAVIIESLKVGAGFLLSDFYPSINLLPVITGALFRIQ

*.*::::**::*:**:::*:****:***:**** ****

k19819_g1_i5 RIHHRLDRLFDTIIKQHKSAA--------KIDDDDDSIEDLVDVLLKVQKDGSLTTETIK

CL2946Contig1 RVHRKFDKLLDGIIKQHRARAAANTTTNGQIVDDDDKYEDFVDVLLKFERDGSLTTANIK

*:*:::*:*:* *****:: * :* ****. **:******.::****** .**

k19819_g1_i5 AVLLNMFTAGTDTSTTAIEWAMSEMIKNPSKLNKAQEEVRKVFDNKGYVEEDKFDELKYL

CL2946Contig1 AVLLDMFLGGTDTSANTVEWAMSELIKNPSELSKAQEEVRRVFDDKGYVDEDKFDELKYL

****:** .*****:.::******:*****:*.*******:***:****:**********

k19819_g1_i5 KLIIKETLRLHPPVPLLIPRMSSQRCEIDGYEIPAKTTVIVNAWALGRDPEYWKDPEKFI

CL2946Contig1 KLIIKETMRLHPALPLLLPRMSSQRCEINGYEIPAGTRVIVNAWALGRDPKYWKDAEKFI

*******:**** :***:**********:****** * ************:**** ****

k19819_g1_i5 PERFEESSIDFKGNNLEYIPFGAGRRMCPGMTFGLANVELPLAMLLYHFDWKMPKGIKNE

CL2946Contig1 PERFEESSHDFHATNLEYIPFGAGRRICPGISFGIANVQLPLSMLLYHFDWKMPNGIKGE

******** **:..************:***::**:***:***:***********:***.*

k19819_g1_i5 DLDMTEAFGVTVKRKHHLHLIPIVKRPLPAR-

CL2946Contig1 DLDMSEAFGITVNRNHHLHLVPTVKRPLSAAA

****:****:**:*:*****:* ***** *

Supplementary Figure S4. Alignment of sequences CYP76AK3.

>CL6853Contig2

MQIFILLSLALVAAWATYSRWSEPRRRSVPPGPPRLPIVGNILQLGPNPHKSFAHLAKTYGPLMSLHLGNQFAVVVSSPEMATEVLQKQGLVFANPFNPVALRVLGHDENSVAMLPSSTATWKKLRRVARERLFSHQALQASQGLRHERLRRLIDRVGECRGAMNVGEATFTTTSNIMFATLFSADFGGGAAASKVLREHVKSFTRYIGLPNISDFFPIFAPLDPQGIKKKVIHHLGGLLEIVSGMIEQRLQERKESNYQKKNDFLETLLDLIQGHEYDLSIEEIKHLCVDLLISGSDTSAATTEWAMVELLLHPDKLAKLKAELKSVIGDQTLVEESDISRLPYLQATIKEVLRYHPAAPLLAPHVAEEETQLDGYLIPKNTKMFVNFWAITRDPTIWKDPERFEPERFLDKDIDFSGQHFELIPFGSGRRICPGMALATRMLPCMVAALCHNFDWELERGNECKRLQREDVFGLALQKKTPLWAIPIKV

>evm.model.scaffold1486.2

MAAARQVPSGAASSADHRQRLAARAEPPQVAGRPRQTLRPTHVPPARYPIRGRGFVAGDGEGSAAEARPCFLHALQRRGGVCPRPRAHVHGLALHQLQPVAKASPHIQRAPLLASSSPGQPTSPAREADQAGRPRQQVLRAGPRHERRRSHLHHHVQPHVRHALFHRSHRIRRRRFRRQKGTEGAVNKLMRVAGAPNVADFFPILAPFDPQGLRRKLTYHLRCLMDLIQTLIDERLQARAALPYHKKNDSLETLLDLVEGQEYDFTTEEIKHMFVDLIIAGSDTSAATTEWAMVELLLHPDKLEKLKAELKSVLGEQSIVDESDISRLPYLQATVKEVLRCHPAAPLLAPHAAEVETHINGYVIPKNAKMFINVWAISRDRSIWKNPESFEPERFLDNGIDFGGHHFELIPFGSGRKVCPGMPLASRMLHCMVATLCHNFDWKLEQGTESKQLQRQDVFGLVLQKKSPLWAIPIKL

CLUSTAL O (1.2.4) multiple sequence alignment

CL6853Contig2 MQIFILLSLALVAAWAT--------YSRWS-------EPRR--RSVPPGPPRLPIVGNIL

evm.model.scaffold1486.2 -----MAAARQVPSGAASSADHRQRLAARAEPPQVAGRPRQTLRPTHVPPARYPIRGRGF

: : * : *: : : .**: * . * * ** *. :

CL6853Contig2 QLGPNPHKSFAHLAKTYGPLMSLHLGNQFAVVVSSPEMATEVLQKQGLVFANPFNPVA--

evm.model.scaffold1486.2 VAGDGEGSAA-----------------------EARPCFLHALQRRGGVCPRPRAHVHGL

* . .: .: ..**::* * .* *

CL6853Contig2 -LRVL---GHDE---NSVAMLPSSTAT-----WKKLRRVARERLFSHQALQASQGLRHER

evm.model.scaffold1486.2 ALHQLQPVAKASPHIQRAPLLASSSPGQPTSPAREADQAGRPR---QQV--LRAGPRHER

*: * .: . : . :* **: :: :..* * :*. * ****

CL6853Contig2 LRRLIDRVGECRGAMNVGEATFTTTSNIMFATLFSADFGGGAAASKVLREHVKSFTRYIG

evm.model.scaffold1486.2 RRSHLHHHV----QPHVRHALFHRSHRI-RRRRFRRQK--------GTEGAVNKLMRVAG

* :.: :* .* * : .* * : . *:.: * *

CL6853Contig2 LPNISDFFPIFAPLDPQGIKKKVIHHLGGLLEIVSGMIEQRLQERKESNYQKKNDFLETL

evm.model.scaffold1486.2 APNVADFFPILAPFDPQGLRRKLTYHLRCLMDLIQTLIDERLQARAALPYHKKNDSLETL

**::*****:**:****:::*: :** *::::. :*::*** * *:**** ****

CL6853Contig2 LDLIQGHEYDLSIEEIKHLCVDLLISGSDTSAATTEWAMVELLLHPDKLAKLKAELKSVI

evm.model.scaffold1486.2 LDLVEGQEYDFTTEEIKHMFVDLIIAGSDTSAATTEWAMVELLLHPDKLEKLKAELKSVL

***::*:***:: *****: ***:*:*********************** *********:

CL6853Contig2 GDQTLVEESDISRLPYLQATIKEVLRYHPAAPLLAPHVAEEETQLDGYLIPKNTKMFVNF

evm.model.scaffold1486.2 GEQSIVDESDISRLPYLQATVKEVLRCHPAAPLLAPHAAEVETHINGYVIPKNAKMFINV

*:*::*:*************:***** **********.** **:::**:****:***:*.

CL6853Contig2 WAITRDPTIWKDPERFEPERFLDKDIDFSGQHFELIPFGSGRRICPGMALATRMLPCMVA

evm.model.scaffold1486.2 WAISRDRSIWKNPESFEPERFLDNGIDFGGHHFELIPFGSGRKVCPGMPLASRMLHCMVA

***:** :***:** ********:.***.*:***********::**** **:*** ****

CL6853Contig2 ALCHNFDWELERGNECKRLQREDVFGLALQKKTPLWAIPIKV

evm.model.scaffold1486.2 TLCHNFDWKLEQGTESKQLQRQDVFGLVLQKKSPLWAIPIKL

:*******:**:*.*.*:***:*****.****:********:
